# Fatty acid synthesis therapy-induced senescence (FASTIS) in cancer cells

**DOI:** 10.1038/s41419-026-08992-8

**Published:** 2026-06-17

**Authors:** Sara Verdura, Àngela Llop-Hernández, Travis Van der Steen, José Antonio Encinar, Begoña Martin-Castillo, Elisabet Cuyàs, Ruth Lupu, Javier A. Menendez

**Affiliations:** 1 Program Against Cancer Therapeutic Resistance (ProCURE), Catalan Institute of Oncology, Girona, Spain; 2 Metabolism and Cancer Group, Girona Biomedical Research Institute (IDIBGI), Girona, Spain; 3 Division of Experimental Pathology, Department of Laboratory Medicine and Pathology, Mayo Clinic, Rochester, MN USA; 4 Institute of Research, Development and Innovation in Health Biotechnology of Elche (IDiBE), Universitas Miguel Hernández (UMH), Elche, Spain; 5 Unit of Clinical Research, Catalan Institute of Oncology, Girona, Spain; 6 Mayo Clinic Cancer Center, Rochester, MN USA; 7 Department of Biochemistry and Molecular Biology, Mayo Clinic, Rochester, MN USA

**Keywords:** Breast cancer, Translational research

## Abstract

Therapy-induced senescence (TIS) in cancer cells can be triggered by radiotherapy, chemotherapy, and certain targeted therapeutics. Here, we demonstrate that a new form of TIS, termed fatty acid synthesis therapy-induced senescence (FASTIS), can be induced by pharmacologically targeting de novo lipogenesis. Cancer cells can evade the anti-proliferative effects of clinically relevant inhibitors of core lipogenic enzymes, such as acetyl-CoA carboxylase (ACC) and fatty acid synthase (FASN), by entering in a senescence-like state. FASTIS cancer cells acquire the classical senescence hallmarks, such as cytomorphological remodeling, increased senescence-associated beta-galatosidase (SA-β-gal) activity, activation of cell cycle arrest markers, and hypersensitivity to IFNγ-induced activation of the immune checkpoint PD-L1. mRNA sequencing reveals an FASTIS-associated transcriptomic profile that overlaps between ACC and FASN inhibitors yet differs significantly from that of other mechanistically diverse TIS inducers, including bleomycin, alisertib, doxorubicin, and palbociclib. The FASTIS-encoding transcriptome is characterized by the activation of cholesterol- and acetyl-CoA-related lipogenic pathways, as well as cell-intrinsic innate immune responses. This profile is characterized as highly senescent (≥0.95) by the machine learning–based senescence predictor SENCAN. Mapping the metabolome and lipidome in FASTIS cells reveals a significant sterol lipid enrichment, including substantial increases in intracellular cholesterol levels. Pharmacological blockade of cholesterol synthesis or promotion of lysosomal cholesterol accumulation, prevents or potentiates the occurrence of SA-β-gal+ FASTIS cells, respectively. Cytokine arrays and miR-146a reporter-based screens revealed that the FASTIS-associated secretory phenotype (FASASP) is highly enriched in immunomodulatory factors but not in inflammatory components. FASTIS cancer cells exhibit an increased overall level of mitochondrial priming, making them highly susceptible to targeted senolysis by BCL-xL-targeting BH3 mimetics and cytokine-activated T cells. The FASTIS phenomenon is a therapeutic outcome through which cancer cells adapt to survive clinical-grade lipogenesis inhibitors. The cholesterol-addicted FASTIS fate can be rationally exploited as a collateral sensitivity in “one-two punch” senogenic-(immuno)senolytic strategies.

## Introduction

Cellular senescence is a tumor-suppressive and immunogenic phenomenon that blocks the proliferation of damaged cells, facilitating their immune-mediated clearance [1–4]. Although the ability to evade senescence and subsequent immune surveillance is a hallmark of cancer [5, 6], the antitumor efficacy (or lack thereof) of cancer therapies may be due in part to their ability to re-engage senescence programs in tumor cells. Many common cancer treatments, including radiotherapy, chemotherapy, and some targeted agents, such as CDK4/6 inhibitors, can reactivate senescence in established tumors, leading to profound immune-mediated tumor regression or sensitization to immunotherapy [7–9]. However, the aberrant persistence of therapy-induced senescence (TIS) phenomena in tumor and normal tissues can also cause chronic inflammation and dysfunction of non-cancerous cells, thereby promoting tumor relapse and metastasis, therapy resistance and treatment-related iatrogenic toxicities [10–13].

Not all TIS phenotypes in cancer cells are created equal. We are rapidly learning that the composition of the senescence-associated secretory program (SASP) is a/the effector arm that largely underlies the beneficial versus deleterious effects of TIS in cancer patients [4]. A highly inflammatory and pro-angiogenic SASP can induce a pro-tumorigenic milieu that may help TIS cancer cells to enhance their migratory phenotype, increase their therapeutic resistance, and evade immune elimination. Accordingly, some TIS cancer cells re-enter the cell cycle, promote cancer stemness, and ultimately contribute to tumor aggressiveness [14, 15]. This type of SASP is also likely to contribute to chemotherapy-induced adverse effects in normal tissues, including myelosuppression, fatigue, cardiovascular dysfunction, and the development of secondary malignancies [9–11]. Conversely, some SASP types can activate anti-tumor immunity by enhancing antigen presentation, cytotoxic T-cell infiltration, and natural killer/T-cell activation, thereby reducing tumor regression [2–4, 16]. In this setting, the TIS-associated secretome itself is an underappreciated form of immuno-oncology that can induce robust anti-tumor responses even in the face of cytostatic targeted therapies [4, 17]. Furthermore, TIS can render cancer cells permanently or transiently vulnerable to synthetic lethal approaches targeting acquired senescent vulnerabilities with an increasing number of senotherapies, a two-step synergistic working model often called “one-two punch” [18–21]. However, increased sensitivity to so-called senolytics is not necessarily a core feature of all TIS phenotypes, as some TIS cancer cells are completely refractory to widely used senolytic drugs, such as the BH3 mimetic ABT-263/navitoclax [22–26]. Therefore, it is of paramount importance to understand which TIS agents can maximize components involved in (anti-tumor) immunity and sensitization to senotherapies while minimizing senescence traits involved in (pro-tumor) inflammatory responses and resistance to senotherapies.

Over the past 30 years, there has been an increasing recognition of metabolic disturbances in tumor cells [27–30]. While this has sparked renewed interest in targeting tumor metabolism with next-generation drugs, progress has been limited. Only a few metabolism-based cancer drugs have been approved or are in clinical trials [31, 32]. We hypothesized that novel forms of “metabolic TIS” might constitute understudied barriers to the successful incorporation of targeted metabolic drugs in the era of precision oncology. Here, we specifically investigated whether a TIS-like phenomenon might occur following the pharmacological inhibition of de novo fatty acid synthesis (FASyn), a well-established facet of cancer-associated lipid metabolism remodeling largely controlled by the lipogenic enzymes acetyl-CoA carboxylase (ACC) and fatty acid synthase (FASN) [33–37] (Fig. S1A). We used two clinically relevant FASynis: ND-646 (Nimbus Therapeutics), an orally bioavailable, small-molecule ACC inhibitor that allosterically disrupts ACC dimerization and activity by binding to the AMPK phosphorylation site of ACC [38–41]; and TVB-3166 (Sagimet), an analog of the reversible, selective, orally available FASN inhibitor TVB-2640 –the only FASyni in clinical trials– that blocks the consumption of NADPH by the ketoacyl reductase domain of FASN [42–45] (Fig. S1A).

Using a combination of senescence-associated hallmarks, mRNA sequencing (mRNA-seq), secretomics, metabolomics/lipidomics, functional profiling of the mitochondrial apoptotic priming, and (immuno)senolytic assays (Fig. S1B), we now provide evidence that the pharmacological depletion of endogenously de novo produced FAs is a metabolic stress capable of promoting a senescent fate in lipogenesis-addicted breast cancer cells. Because the senescence program triggered by FASyn inhibitors (FASyins) differs, at least in part, from those activated by mechanistically distinct TIS inducers that cause oxidative, mitotic, genotoxic, or replicative stress, we named this phenomenon FASyn therapy-induced senescence (FASTIS). The discovery and molecular description of FASTIS reveal an unforeseen therapeutic outcome through which cancer cells adapt to survive the first generation of potent, selective, and clinically available FASynis. Furthermore, we propose a therapeutic framework in which FASTIS can be used as a therapeutically exploitable intermediate cell fate of FASynis for the development of “one-two punch” senogenic-(immuno)senolytic strategies.

## Results

### Targeted inhibition of FASyn activates a senescence-like program in breast cancer cells

A sensitivity gradient to the anti-proliferative and anti-clonogenic effects of ND-646 and TVB-3166 was observed in three breast cancer (BC) cell models that naturally express different levels of the lipogenic enzymes ACC and FASN (SKBR3 » MCF-7 > MDA-MB-231) [46–49]. In the short-term plaque assay, both ND-646 and TVB-3166 reduced cell expansion, indicating a predominantly antiproliferative effect, which was more pronounced in SKBR3 and MCF-7 cells (Fig. S2A). Suppression of long-term clonogenic outgrowth, which was also most evident in SKBR3 cells, also suggests that FASN/ACC inhibition impairs long-term survival and colony-forming capacity Fig. S2B). However, even in the most sensitive model, the persistence of a significant residual cell population indicates that FASN/ACC inhibitor-induced growth arrest does not result in extensive cell death, which is consistent with previous reports [45, 49–52]. This prompted us to explore whether the surviving cells adopt senescence-like phenotypic and molecular features.

**Fig. 1:**
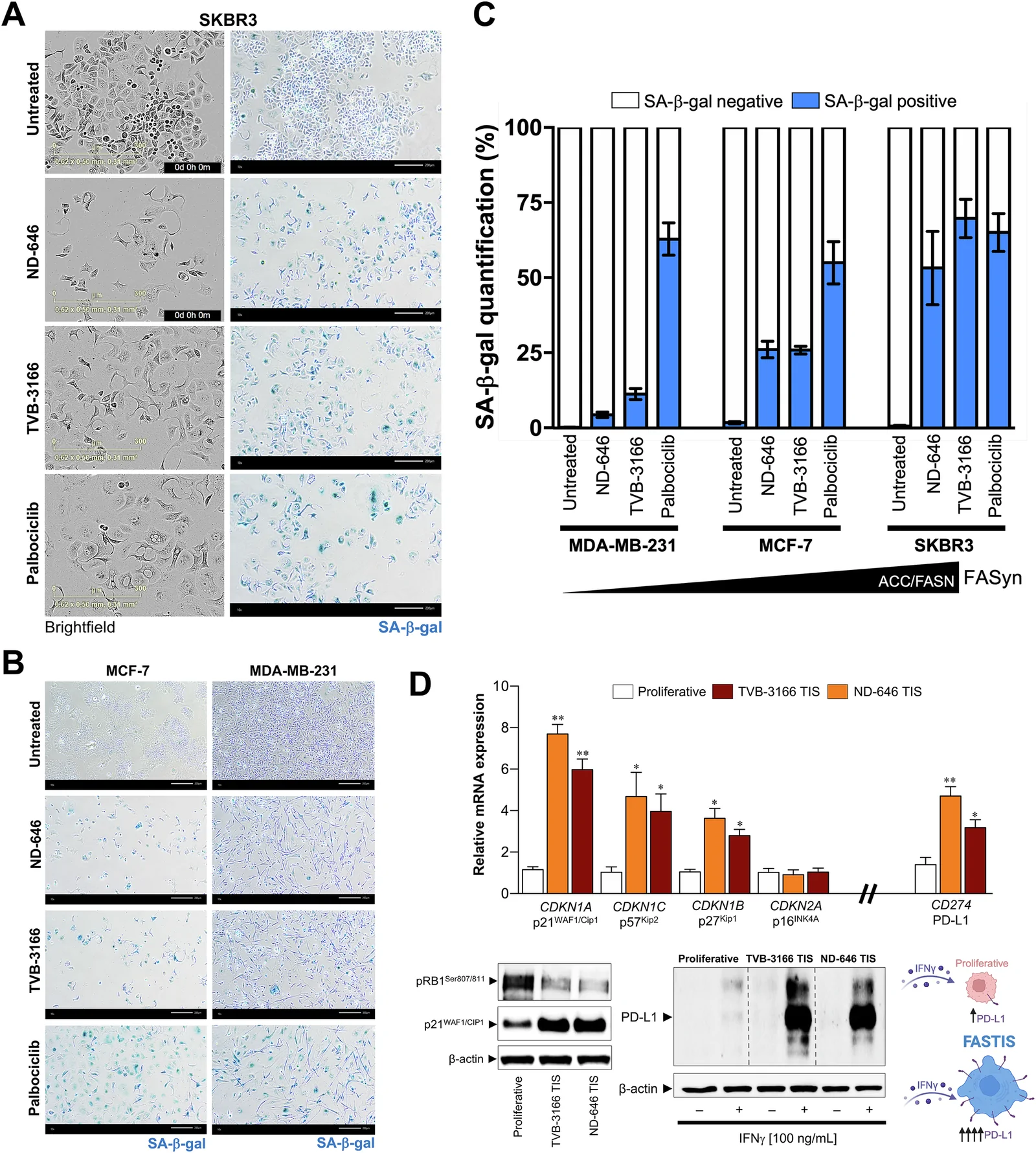
Targeted inhibitors of de novo fatty acid synthesis induce senescence in breast cancer models. **A** *Left*. Representative brightfield and SA-β-gal staining images (*n* = 5) of SKBR3 breast cancer cells untreated or treated with the ACC inhibitor ND-646 (1 μmol/L), the denifanstat (TBV-2640)-like FASN inhibitor TVB-3166 (1 μmol/L), and the CDK4/6 inhibitor palbociclib (5 μmol/L) for 7 days (scale bar: 300 μm, brightfield; 200 μm, SA-β-gal). **B** Representative SA-β-gal staining images (*n* = 5) of MCF-7 and MDA-MB-231 breast cancer cells, untreated or treated with ND-646 (1 μmol/L), TVB-3166 (1 μmol/L), and palbociclib (5 μmol/L) for 7 days (scale bar: 200 μm). **C** At least 2000 cells were quantified from senescence-associated β-galactosidase (SA-β-gal) experiments, and the results are shown as percentages of negative (white) and positive (blue) cells. Data represent the mean ± S.D. of at least five independent experiments performed in triplicate with MDA-MB-231, MCF-7, and SKBR3 cells, which demonstrate graded dependency on ACC/FASN expression and FASyn for cell proliferation and survival. **D** *Top*. Relative mRNA levels of *CDKN1A* (p21), *CDKN1C* (p57), *CDKN1B* (p27), *CDKN2A* (p16), and *CD274* (PD-L1) evaluated by qRT-PCR in SKBR3 cells treated with ND-646 (1 μmol/L) and TBV-2640 (1 μmol/L) for 7 days. Values represent the fold-change expression (mean ± SD) relative to untreated proliferative controls (*n* = 3). Statistically significant differences between the proliferative and TIS phenotypes are shown (* *p* < 0.05; **p < 0.005) using ANOVA analysis. *Bottom*. Expression levels of phospho-RB^Ser807/Ser811^ and p21^WAF1/Cip1^ were detected by immunoblotting in whole cell lysates of proliferative (untreated) and ND-646-/TVB-3166-treated SKBR3 cells (7-d) using specific antibodies and β-actin as loading control. Figure also shows representative immunoblot analysis (*n* = 3) of PD-L1 expression in proliferative (untreated) and ND-646-/TVB-3166-treated SKBR3 cells, either exposed or not exposed to IFNγ (100 nmol/L) for 24 h.

First, we tested whether the residual post-treatment population following exposure to FASynis exhibits two well-established senescence hallmarks: senescence-associated (SA) cytomorphological remodeling and increased lysosomal mass with accompanying acidification dysfunction (Fig. 1). The latter promotes enhanced SA-β-galactosidase (SA-β-gal) activity. SKBR3, MCF-7, and MDA-MB-231 breast cancer cell models were treated with high doses (1 μmol/L) of ND-646 and TVB-3166 for 7 days, after which the cells were imaged using a live-cell imaging platform (IncuCyte^®^) and fixed and stained colorimetrically for SA-β-gal activity. We used the proliferation blocker palbociclib, a cyclin-dependent kinases 4 and 6 (CDK4/6) inhibitor that induces a strong senescence response via replicative stress in breast cancer cells, as a positive control [17, 53–55].

HER2 + SKBR3 cells, the archetypal model of ACC/FASN overexpression in breast cancer [46–48, 56, 57], acquired several changes characteristics of senescent cell morphology in response to ND-646 and TVB-3166, including hypertrophic and more flattened morphology, irregular membrane contours with cytoplasmic protrusions and fewer contacts with surrounding cells, increased cytoplasmic granularity and enlarged intracellular vacuoles, enlarged and often irregularly shaped nuclei, or even multinucleated morphology [58–60] (Fig. 1A). This profound cytomorphological remodeling was less pronounced but still evident in moderate ACC/FASN-expressing ER + /HER2- MCF-7 cells. Low-ACC/FASN-expressing ER-/HER2- MDA-MB-231 cells acquired a noteworthy thin, elongated, spindle-shaped fibroblast-like morphology in response to ND-646 and TVB-3166 (Fig. 1B).

Induction of SA-β-gal occurred in response to ND-646 and TVB-3166, but was highly dependent on the ACC/FASN status of the breast cancer cells. Up to 65-70% of the ACC/FASN-overexpressing SKBR3 cells exhibited high SA-β-gal activity (SA-β-gal^+^ cells) on day 7 after treatment with 1 μmol/L ND-646 and TVB-3166 (Fig. 1C). The percentages of SA-β-gal^+^ cells reached ~25% in moderate ACC/FASN-expressing MCF-7 cells. ACC/FASN-low MDA-MB-231 cells were less senescence-responsive to FASynis, but still significant percentages of SA-β-gal-positive cells (up to 10%) were observed in response to ND-646 and TVB-3166. Palbociclib induced a similar increase in the number of SA-β-gal-positive cells (up to 45-55%) in all breast cancer models regardless of their lipogenic phenotype (Fig. 1C).

To confirm that the senescence-like phenotype induced by FASynis was not specific to ND-646/TVB-3166 drugs or SKBR3 breast cancer cells, we exposed SKBR3 and BT-474 cells –an additional model of lipogenic breast cancer cells [46, 56]– to the macrocyclic myxobacterial natural product soraphen A, which disrupts ACC enzyme dimerization to inhibit ACC enzymatic activity analogous to ND-646 [61, 62], and C75, a cerulenin-derived semisynthetic compound that interferes with the binding of malonyl-CoA to the β-ketoacyl synthase domain of FASN [63, 64]. We confirmed the presence of SA cytomorphological remodeling and increased SA-β-gal activity in soraphen A- and C75-treated SKBR3 and BT-474 cells (Fig. S3).

We then profiled whether the accumulation of SA-β-gal-positive breast cancer cells in response to ND-646/TVB-3166 was accompanied by the upregulation of key CDK inhibitors (CDKis), such as the CDK2is p21^WAF1/Cip1^ (*CDKN1A*), p27^Kip1^ (*CDKN1B*), and p57^Kip2^ (*CDKN1C*), and/or the CDK4/6i p16^INK4A^ (*CDKN2A*), which are known to become activated in senescent cells. Quantitative real-time PCR (qRT-PCR) gene expression analysis revealed that SA-β-gal-positive cells induced by both FASynis dramatically upregulated (by ∼6 to 7-fold) p21^WAF1/Cip1^ and p57^Kip2^ (by ∼5 to 6-fold) mRNAs, whereas p16^INK4A^ was not upregulated (Fig. 1D). Although less pronounced, the ND-646- and TVB-3166-induced senescent-like phenotype was also accompanied by a significant upregulation of p27^Kip1^ (by ∼2 to 4-fold). The observed up-regulation of CDKis, such as p21^WAF1/Cip1^ at the protein level was paralleled by cell cycle arrest, as an almost complete loss of Ser-807/811 retinoblastoma (RB) phosphorylation, a hypophosphorylated and active state of RB that inactivates E2 family transcription factors to arrest cell cycle progression, was observed on day 7 after treatment with FASynis (Fig. 1D).

Previous studies have shown that senescence induced by radiation therapy, chemotherapy (e.g., doxorubicin) and cell cycle inhibitors (e.g., palbociclib) can increase the expression levels of immune checkpoints, such as PD-L1 (*CD274*) [65–68]. A 7-day incubation with ND-646 and TVB-3166 slightly but significantly up-regulated *PD-L1* mRNA expression (by ∼3-fold), while no apparent effect on constitutive PD-L1 protein expression was detected (Fig. 1D). In contrast, immunoblotting procedures demonstrated that IFNγ-induced PD-L1 protein expression was dramatically increased in ND-646- and TVB-3166-induced senescent-like cells compared to proliferative counterparts (Fig. 1D).

Taken together, these results demonstrate that FASynis can induce a senescence-like state in breast cancer cells. This state is characterized by cytomorphologic remodeling, SA-β-gal positivity, cell cycle arrest, and hypersensitivity to IFNγ-induced up-regulation of the immune checkpoint PD-L1. We termed this phenomenon FASyn therapy-induced senescence (FASTIS).

### The stability of the FASTIS phenotype requires continuous blockade of FASyn

Senescence is classically defined as the terminal cessation of cell division. However, entry into a radiation-, chemotherapy-, and targeted therapy-induced TIS phenotype is not necessarily a permanent endpoint, but rather a continuum of variable strength and depth towards a full-blown arrest state, dependent on potentially transient senescence-associated maintenance mechanisms that may allow cell cycle re-entry if not continuously provided [69–72].

**Fig. 2:**
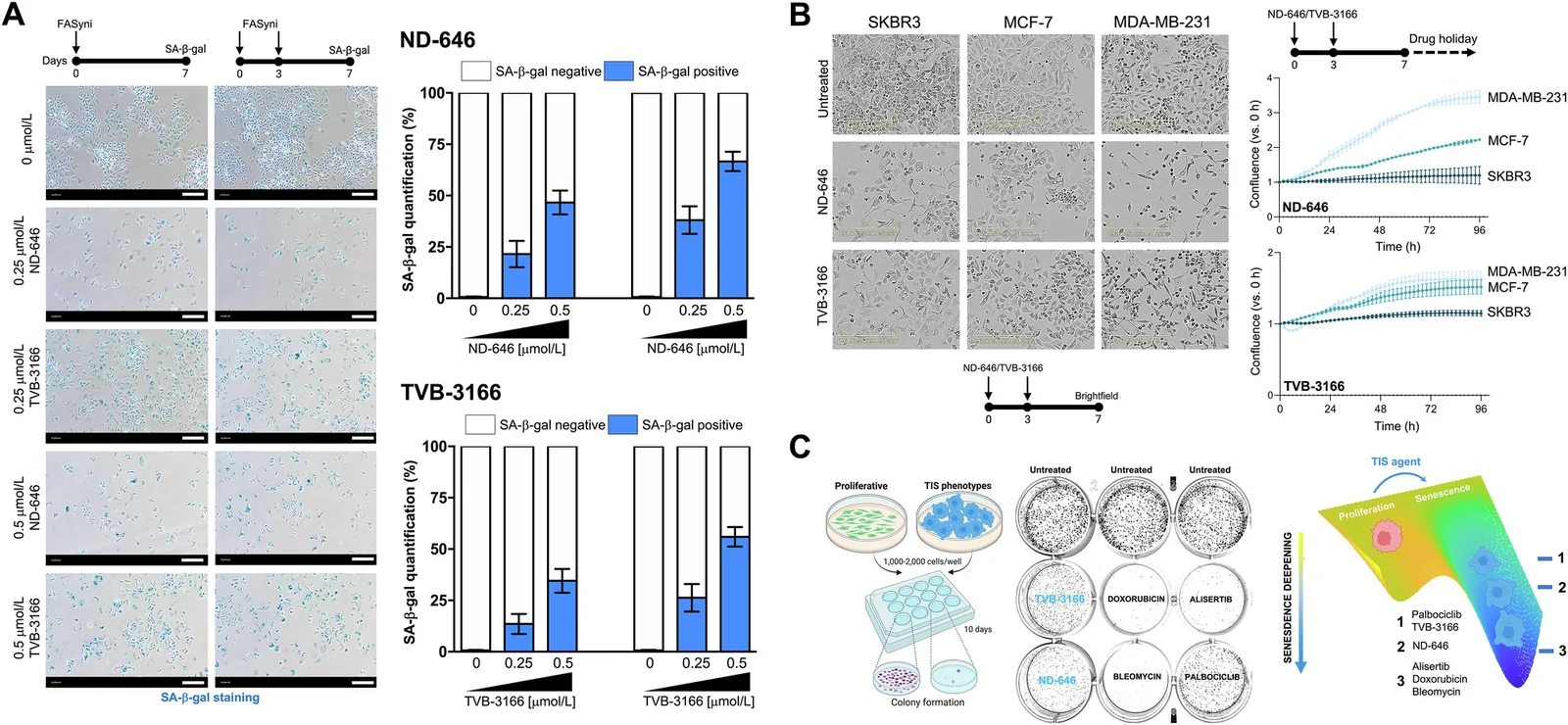
Continuous exposure to fatty acid synthesis inhibitors increases the stability of the senescent phenotype. **A** *Top*. Representative images (*n* = 3) of SA-β-gal staining in SKBR3 breast cancer cells untreated or treated with graded concentrations of ND-646 (0.25 and 0.5 μmol/L) and TVB-3166 (0.25 and 0.5 μmol/L) either for 7 days without drug replenishment or with drug replenishment on day 3 (scale bar: 200 μm). *Bottom*. Figure shows quantification of at least 2000 cells from SA-β-gal staining experiments, shown as percentages of negative (white) and positive (blue) cells. Data represent the mean ± S.D. of at least three independent experiments performed in triplicate. **B** Recovery of proliferation potential after exposure to FASynis was evaluated by monitoring confluence (vs. 0 h) after releasing ND-646- and TVB-3166-treated cells for 7 days into a drug-free, re-fed medium for an additional 4 days (“drug holiday”). Representative brightfield images on the left illustrate cellular morphology at the end of the treatment period. Images and confluence data were obtained using the IncuCyte^®^ S3 Live-Cell Analysis System. **C** *Left*. Recovery of clonogenic potential after acquisition of the FASTIS phenotype was evaluated by reseeding untreated and TIS SKBR3 cells (1000–2000 cells/well) under drug-free conditions. After 10 days, cell growth was monitored by crystal violet staining. Shown are representative images from three technical replicates (*n* = 2; TVB-3166: 1 μmol/L; ND-646: 1 μmol/L; doxorubicin: 50 nmol/L; bleomycin: 20 μmol/L; alisertib: 500 nmol/L; palbociclib: 5 μmol/L). *Right*. The figure illustrates the relationship between the depth of senescence of TIS phenotypes as a function of their proliferative/clonogenic recovery capacity (i.e., the deeper the phenotype, the lower the ability to recover).

To investigate whether the likelihood of breast cancer cells committing to FASTIS depends on the intensity and duration of FASyn blockade, the SA-β-gal-inducing activity of lower doses of FASynis was examined in two senescence induction protocols in ACC/FASN-overexpressing SKBR3 cells, one of 7 days without drug refreshment and another of 7 days with refreshment of FASynis on day 3 (Fig. 2A). We observed a dose-dependent increase in the acquisition of a senescence-like phenotype and SA-β-gal positivity after 7 days of continuous treatment of SKBR3 cells with 0.25 and 0.5 μmol/L concentrations of the ACCi ND-646 and the FASNi TVB-3166 (Fig. 2A). When the drug-containing media were refreshed on day 3, a greater number of SKBR3 cells became senescent, with the extent of SA-β-gal positivity increasing by approximately twofold (from 21% to 38% with ND-646 and from 13 to 26% with TVB-3166) at the lowest concentration used (0.25 μmol/L). At the highest concentration used (0.5 μmol/L), the increase in SA-β-gal positivity was also significantly increased with drug refreshment (from 47% to 67% with ND-646 and from 34% to 56% with TVB-3166; Fig. 2A).

To evaluate the therapeutic effects of triggering FASTIS in lipogenesis-addicted cells, we investigated the capacity of the SA-β-gal+-enriched residual population produced by FASynis to resume proliferation upon cessation of drug administration. SKBR3, MCF7 and MDA-MB-231 cells were treated with ND-646 or TVB-3166 for 7 days, then released into drug-free, re-fed medium and observed for an additional 4 days (Fig. 2B). Longitudinal confluence measurements revealed that the highly lipogenic SKBR3 cells exhibited impaired proliferative recovery. In contrast, the MCF-7 cells displayed partial recovery, and the poorly lipogenic MDA-MB-231 cells demonstrated substantial recovery, particularly after release from ND-646 (Fig. 2B). These data suggest that the long-term proliferative consequences of FASynis largely depend on the initial lipogenic status of cancer cells, thus supporting the notion of FASTIS as a therapeutic outcome in settings involving lipogenesis addiction.

To further assess how close or far the FASTIS phenotype is to the deep attractor of a stable senescence arrest state, we finally tested whether recovery of clonogenic capacity occurs in rare, isolated cells or in most of the residual population containing SA-β-gal^+^ cells generated by FASynis. Untreated and TIS cancer cells induced either by the FASynis ND-646 and TVB-3166 or by well-established, mechanistically distinct TIS inducers, such as bleomycin (an antibiotic that promotes oxidative stress), alisertib (an Aurora A kinase inhibitor that promotes mitotic stress), doxorubicin (a topoisomerase II inhibitor and DNA intercalator that induces DNA damage stress), and palbociclib (a CDK4/6 inhibitor that induces replication stress), were reseeded at low density (1000–2000 cells/well) under drug-free conditions for 10 days to allow colony formation (Fig. 2C). TIS cells exposed to bleomycin, doxorubicin and alisertib showed no clonogenic capacity after 7 days of treatment, demonstrating the stable entry into a full arrest state of their senescent phenotypes. In contrast, palbociclib-induced senescent cells showed a partial but significant recovery of clonogenic potential after drug release. ND-646- and TVB-3166-induced TIS populations exhibited a palbociclib-like behavior, as an observable number of colonies were formed after washout of FASynis. This partial recovery of clonogenicity was less pronounced in the ND-646-induced FASTIS phenotype (Fig. 2C).

Taken together, these results demonstrate that the stability of the FASTIS phenotype requires continuous blockade of FASyn, resulting in a state of arrest similar in severity and senescence deepening to palbociclib TIS, but less pronounced than bleomycin TIS, alisertib TIS, and doxorubicin TIS (Fig. 2C).

### The FASTIS-associated transcriptomic signature is scored as highly senescent by the machine-learning-developed SENCAN classification model

**Fig. 3:**
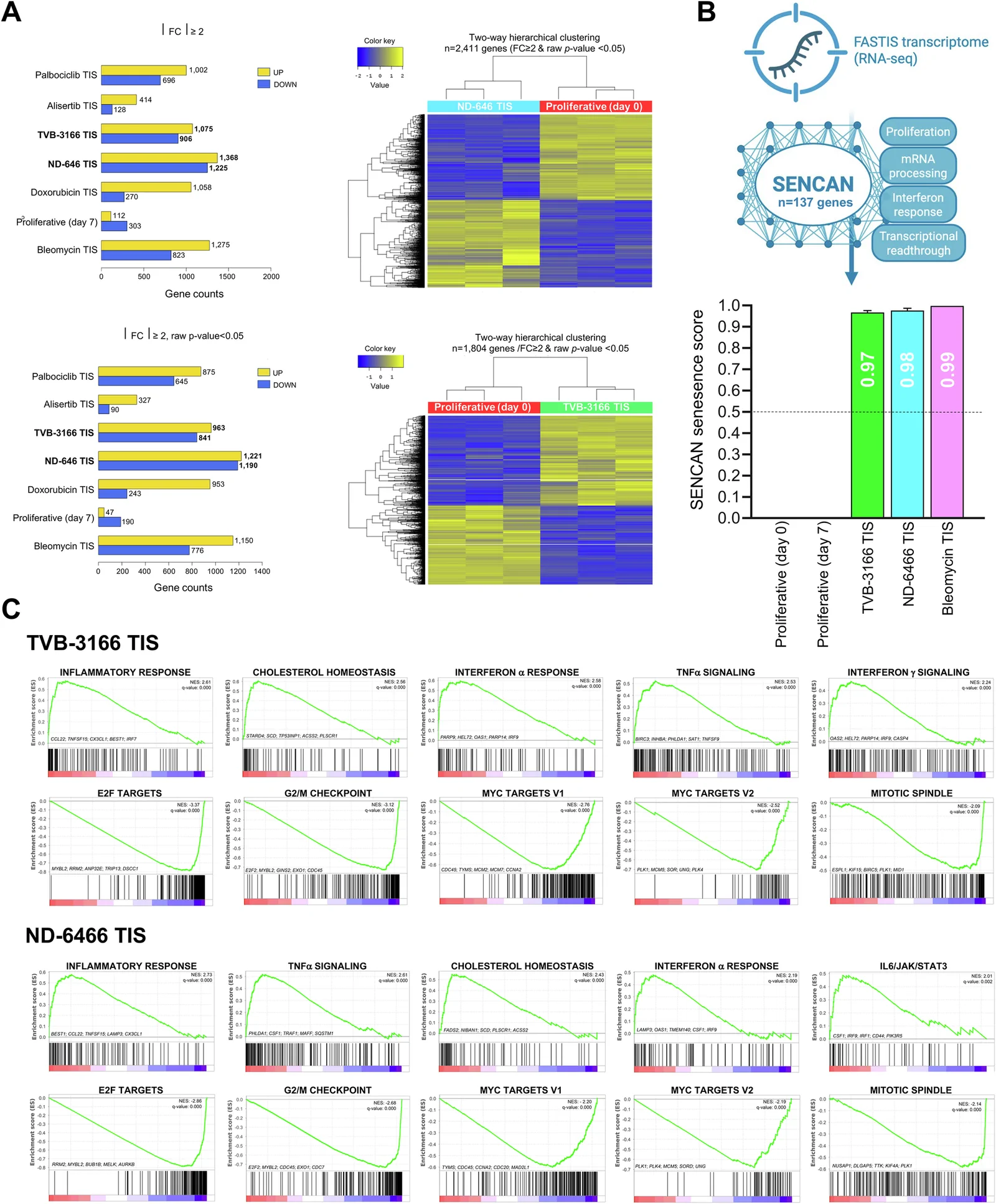
The FASTIS phenotype exhibits a core senescence-associated transcriptomic signature. **A** *Left panels*. DEG results of TIS agents *versus* proliferative controls (day-0) showing the number of up- and down-regulated genes based on either fold-change of the comparison pair (*top*) or fold-change and raw *p*-value (<0.05) of the comparison pair (*bottom*). *Right panels*. Two-way hierarchical clustering of genes and samples' similarity using *z*-score for normalized values (log_2_-based). **B** Senescence scores of processed RNA sequencing data of untreated proliferative controls and TIS phenotypes were calculated by the SENCAN elastic net classifier (*n* = 137 genes) for cancer cell senescence using the available script at https://ccb.nki.nl/publications/cancer-senescence/. **C** Hallmark enrichment analysis showing the strongest positive and negative associations with the TVB-3166 TIS (*top*) and ND-646 TIS (*bottom*) transcriptomic signatures. The GSEA normalized enrichment scores (NES) and FDR-adjusted *p*-values, as well as the top 5 associated genes, are shown for each positively and negatively associated feature.

To better understand the unique versus shared molecular changes underlying the FASTIS phenotype triggered by FASynis, compared with other TIS inducers, we performed mRNA-seq to provide a comprehensive, high-resolution view of the underlying coding transcriptomes. ACC/FASN-overexpressing SKBR3 cells were rendered senescent using ND-646 and TVB-3166 in strict parallel with the TIS inducers bleomycin, alisertib, doxorubicin, and palbociclib. To account for potential artifacts due to cell subpopulations entering a quiescence state to survive nutrient deprivation after 7 days, parallel mRNA sequencing was also performed in untreated (proliferative) cells at days 0 and 7. Compared to the control group of untreated cells at day-0 (fold-change >2, *p* < 0.05), a total of 1221 genes were upregulated in the ND646-induced (day-7) senescent group, while 1190 genes were downregulated. In the TVB-3166-induced (day-7) senescent group, a total of 963 genes were upregulated while 841 genes were downregulated (Fig. 3A). Notably, ND-646- and TVB-3166-induced senescent cells were among the TIS phenotypes with a higher number of significantly altered genes (2411 and 1804 genes, respectively) compared to bleomycin TIS (1926 genes), palbociclib TIS (1520 genes), doxorubicin TIS (1196 genes), and alisertib TIS (417 genes) (Fig. 3A).

We used the SENCAN classification model, an elastic net-based classifier constructed using bulk RNA sequencing data from 13 cancer cell lines induced to senesce by treatment with alisertib and etoposide [73], to measure the probability of senescence in TVB-3166- and ND-646-treated cells based on RNA-seq data. The transcriptome of ND-646 and TVB-3166 TIS cells was scored as highly senescent (≥0.95) as the bleomycin-induced TIS (0.98) by the SENCAN classifier (Fig. 3B). Gene set-based analysis of the estimated gene associations with ND-646 TIS showed the strongest (nominal *p* < 0.001, FDR *q*-value > 0.1) positive correlations with hallmarks, such as inflammatory response, TNFα signaling, cholesterol homeostasis, interferon α response, and IL6 JAK STAT3 signaling (Fig. 3C). In the case of TVB-3166 TIS, the strongest positive correlations also occurred with features, such as inflammatory response, interferon α response, cholesterol homeostasis, TNFα signaling, and interferon γ response. The strongest negative associations with both ND-646 and TVB-3166 corresponded to E2F targets, G_2_/M checkpoint, MYC targets (V1 and V2), and mitotic spindle (Fig. 3C).

Interrogation of the gene expression patterns of ND-646 and TVB-3166 TIS breast cancer cells using the SENCAN classifier transcriptionally confirmed the activation of a senescence-like transcriptional program in FASTIS breast cancer cells, which was significantly enriched for upregulation of immune clearance-related pathways and downregulation of cell cycle mitotic pathways.

### The FASTIS-encoding transcriptome has distinct functional features overlapping with other TIS phenotypes

**Fig. 4:**
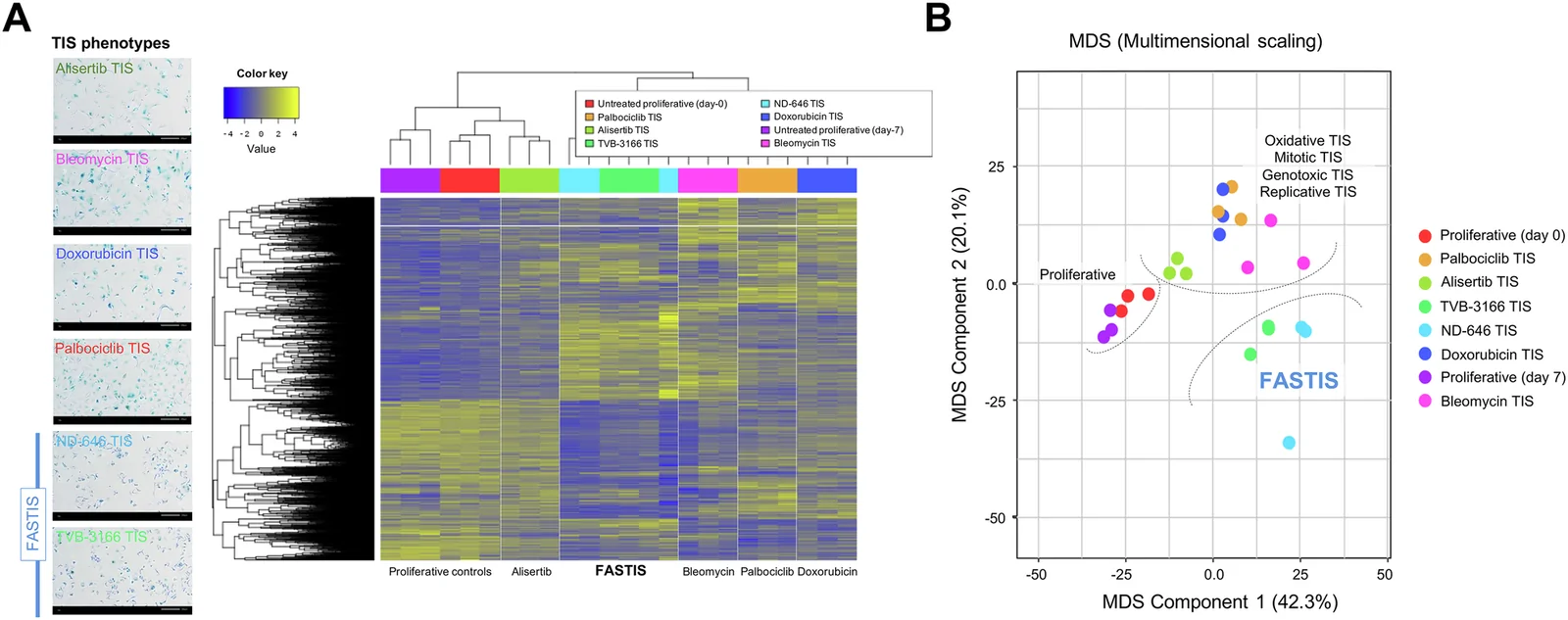
The FASTIS-associated transcriptome is distinctly segregated from other TIS phenotypes. **A** *Left*. Representative images (*n* = 5) of SA-β-gal staining in alisertib, bleomycin, doxorubicin, palbociclib, ND-646, and TVB-3166 TIS phenotypes in SKBR3 breast cancer cells (scale bar: 200 μm). *Right*. Heat map of the two-way hierarchical clustering of TIS phenotypes using z-score (log_2_-based) normalized values (3496 genes satisfying the fold-change and raw *p*-value. **B** Multidimensional scaling (MDS) plot of the similarity between TIS transcriptomes using rlog-transformed values and two principal components. This approach preserves the degree of similarity between samples so that distances correspond to differences in the biological coefficient of variation, allowing the identification of outlier samples and the detection of similar expression patterns across groups of samples.

To determine whether the FASTIS-encoding transcriptome is distinct from other TIS phenotypes, we first analyzed the above RNA-seq data using unsupervised hierarchical clustering and dimensionality reduction analyses (Fig. 4A). When a heatmap was generated to include 3946 genes satisfying the ∣log2, (fold-change) ∣ > 2, *p* < 0.05 threshold, the results showed that the ND-646 and TVB-3166 TIS groups were closer in distance to each other than to untreated day-0 cells, day-7 proliferative cells, and all other TIS phenotypes (Fig. 4A). Multidimensional scaling (MDS) analysis (Fig. 4B) and principal component analysis (PCA; Fig. S4) supported a discriminating separation of ND-646 and TVB-3166 TIS groups from the groups of other TIS inducers.

Next, we classified the differentially expressed (DE) mRNAs identified in the ND-646 and TVB-3166 groups into enriched gene ontology (GO) categories. The three ontology categories (biological processes [BP], cellular component [CC], and molecular function [MF]) were annotated, and the top 20 significantly enriched categories within each ontology were described (Figs. S5–S7). The top BP clustered categories of ND-646 and TVB-3166 TIS were enriched in genes involved in cellular response to stimulus, cell cycle process, chromosome segregation, and organelle fission. The cytoplasm, but also the chromosomal region, spindle, and microtubule, were the CC clustered categories more significantly enriched in ND-646 and TVB-3166 TIS. Protein binding, including tubulin binding, was the MF category more highly enriched in ND-646 and TVB-3166 TIS (Figs.S5). An overall GO analysis including all TIS agents revealed that ND-646 and TVB-3166 TIS shared cell cycle and chromosome segregation related BP categories with palbociclib and bleomycin TIS, but did not overlap with any of the BP categories characteristic of alisertib and doxorubicin TIS (Fig. S8). While cytoplasm was the CC category commonly shared by all TIS phenotypes, ND-646 and TVB-3166 TIS shared enrichment for chromosomal and spindle CC categories with palbociclib and bleomycin TIS, which was not observed in alisertib and doxorubicin TIS (Fig. S8). While protein binding was the MF category commonly shared by all TIS phenotypes, ND-646 and TVB-3166 TIS shared enrichment for the microtubule binding MF categories with palbociclib and bleomycin TIS, which was not observed in alisertib and doxorubicin TIS (Fig. S8).

Taken together, these results demonstrate that the FASTIS phenotype induced by ND-646 and TVB-3166 has a distinct transcriptomic signature that is functionally enriched for mitotic cell cycle, protein binding, and spindle/chromosome segregation processes.

### The FASTIS-encoding transcriptome is enriched for features of lipid homeostasis

**Fig. 5:**
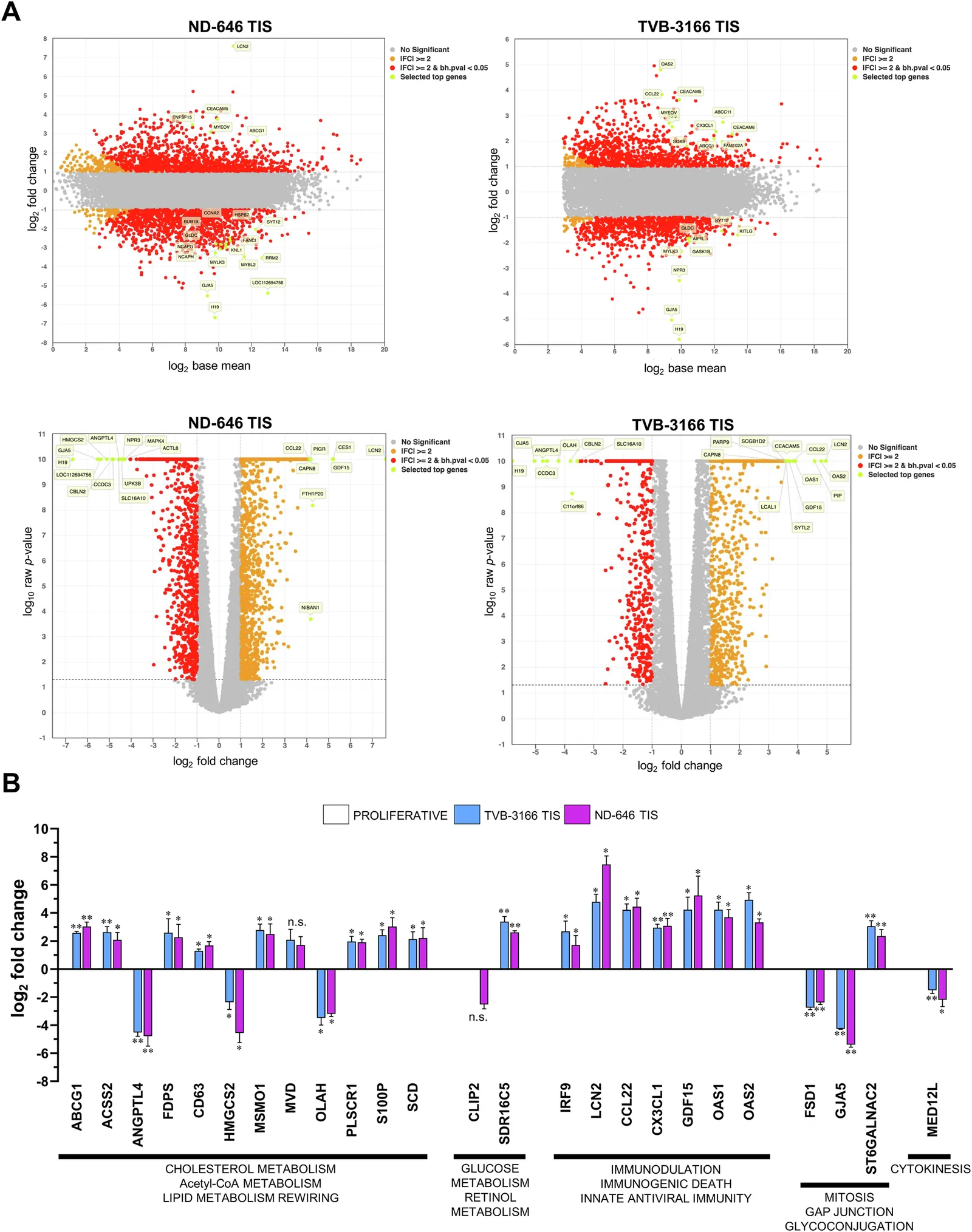
The FASTIS-associated transcriptome included genes involved in lipid metabolism and innate immune response. **A**. *Top*. MA plots showing the overall average expression level between the log_2_ fold-changes (X-axis as the mean of the normalized counts and Y-axis as the log_2_ fold-changes). Selected top genes are highlighted. *Bottom*. Volcano plots showing the log_2_ fold-change and *p*-value obtained by comparing the mean for each group (X-axis: log_2_ fold change, Y-axis: -log_10_ *p*-value). **B**. To validate the accuracy of RNA-seq transcriptomic data, qRT-PCR experiments were performed in a selected group of genes from Tables 1 and 2. The values represent the log2 fold-change values of gene expression of ND-646 and TVB-3166 TIS cells relative to untreated proliferative controls. Statistically significant differences between the proliferative and TIS phenotypes are shown (* *p* < 0.05; **p < 0.005) using ANOVA analysis.

We then analyzed differentially expressed mRNAs to identify unique FASTIS-associated gene expression patterns compared to other TIS phenotypes. First, MA and Volcano plots were generated to provide a graphical representation of the ND-646 TIS- and TVB-3166 TIS-associated DEGs (Fig. 5A). Genes encoding the carcinoembryonic glycoantigen *CEACAM5*, the lipid efflux pump *ABCG1*, and the super-enhancer *MYEOV* were among the top up-regulated genes in the MA plots of the ND-646/TVB-3166-induced FASTIS phenotype (Fig. 5A, *left*). Genes encoding the long non-coding RNA *H19*, the gap junction protein *GJA5*, and the glycine decarboxylase complex (*GLDC*) were among the top down-regulated in the MA plots of the ND-646/TVB-3166-induced FASTIS phenotype (Fig. 5A, *left*).

The top up-regulated genes in the Volcano plots of the ND-646/TVB-3166-induced FASTIS phenotype genes included genes encoding lipocalin-2 (*LCN2*), a hypoxia marker that becomes up-regulated by the TIS SASP to increase breast cancer cell plasticity [74], the stress-induced immunoregulatory cytokine growth differentiation factor 15 (*GDF15*) [75, 76], the cysteine-protease *CAPN8*, and the immunomodulatory chemokine *MDC/CCL22* (Fig. 5A, *right*). Hyperactivation of the genes encoding the 2′,5′-oligoadenylate synthetases *OAS1* and *OAS2* –interferon-stimulated antiviral genes (ISGs) that are activated as a defense mechanism against cytoplasmic RNA in senescent cells [72, 77]– appeared to occur differentially in TVB-3166 (but not in ND-646) FASTIS breast cancer cells. In addition to *H19* and *GJA5/Cx40*, genes encoding the lipogenic/adipogenic secretory factor fat/vessel-derived secretory protein *Favine*/*CCDC3*, and the angiogenic angiopoietin-like 4 (*ANGPTL4*) factor, were among the top down-regulated genes in the Volcano plots of the ND-646/TVB-3166-induced FASTIS phenotype (Fig. 5A, *right*).

**Fig. 6:**
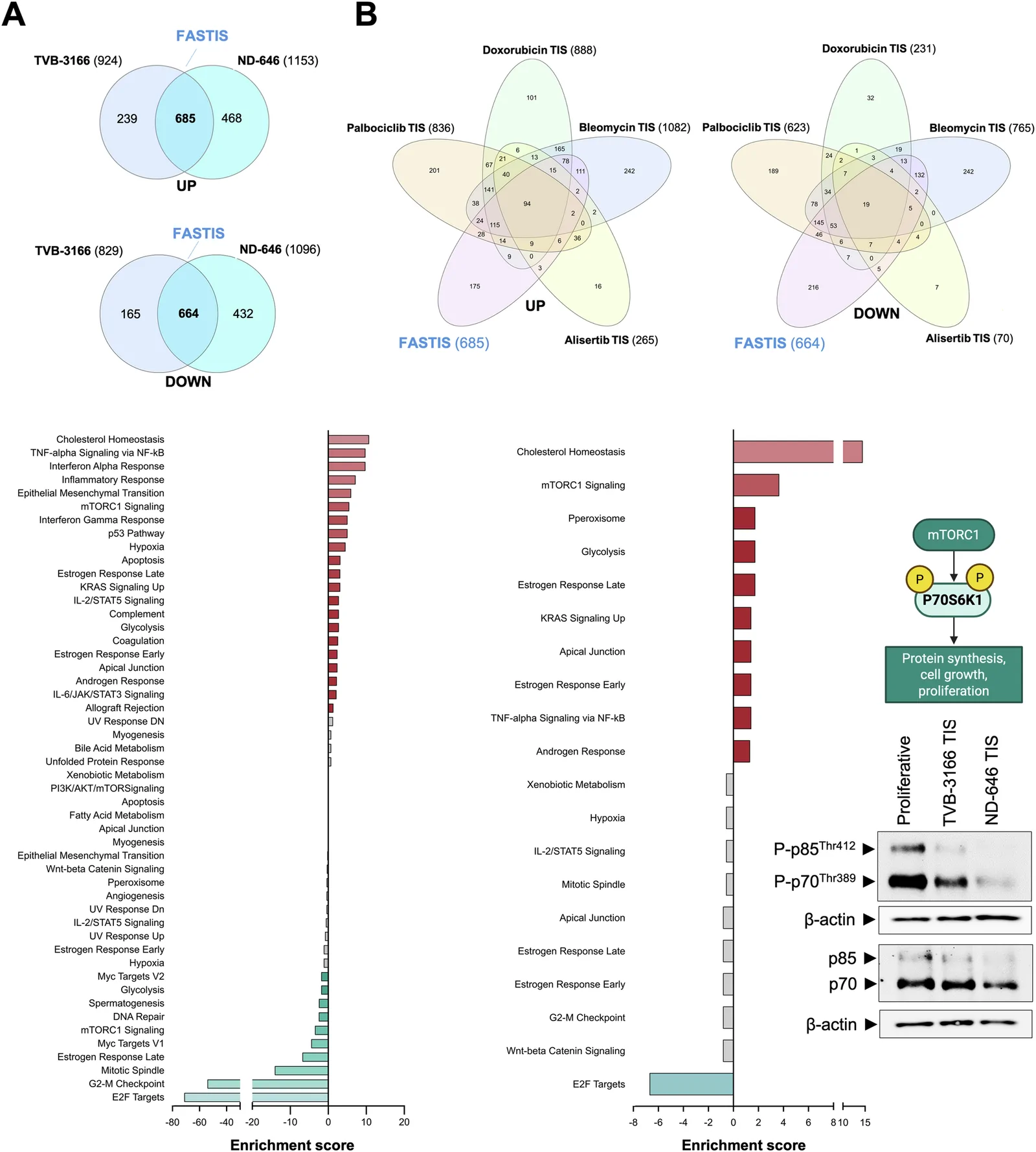
The FASTIS-associated transcriptome exhibits features related to cholesterol homeostasis. **A** *Top*. Venn diagrams showing unique and common up- and down-regulated differentially expressed genes (DEGs) for the TVB-3166 *vs*. ND-646 transcriptome comparisons. *Bottom*. The core set of identified FASTIS DEGs were analyzed for enriched pathways using the EnrichR package (v3.2) and sorted by *p*-value ranking. **B** *Top*. Venn diagrams showing unique and common up- and down-regulated DEGs for all of the TIS transcriptomic comparisons. *Bottom*. The unique set of identified FASTIS DEGs vs. all other TIS phenotypes was analyzed for enriched pathways using the EnrichR package (v3.2) and sorted by *p*-value ranking. A representative immunoblot (*n* = 3) shows phospho-p85^Thr412/Thr389^ S6K1 and total p85/p70 S6K1 in proliferative A549 cells and in TVB-3166- and ND-646 TIS derivatives, with β-actin serving as the loading control.

Second, we defined a FASTIS transcriptomic signature as the 1349 DEGs that were commonly altered (685 up-regulated and 664 down-regulated) in the corresponding signatures of ND-646 and TVB-3166 TIS (Fig. 6A). Functional enrichment analysis of the FASTIS DEGs using the EnrichR database revealed top-enriched GO terms, such as cholesterol homeostasis, TNFα signaling and IFNα response, while top-depleted GO terms included mitotic spindle, G_2_/M checkpoint and especially E2F target genes. As shown in the Venn diagram, a total of 175 genes were exclusively upregulated, and 216 genes were exclusively downregulated in the FASTIS signature compared to other TIS phenotypes. The list of the exclusive top 20 up-regulated genes of the FASTIS signature (Table 1) included genes encoding the calcium-binding protein *S100P*, a key modulator of cell motility during hypoxia that enables TIS onset and rewires lipid metabolism to alter ferroptosis susceptibility [78, 79], the short-chain dehydrogenase/reductase family 16 C member 5 (*SDR16C5*), the sialyltransferase and breast cancer metastasis suppressor *ST6GALNAC2* [80], or the pyruvate dehydrogenase phosphatase *PDP1*. In addition to *ANGPTL4*, the list of the exclusive top 20 down-regulated genes of the FASTIS signature (Table 2) included genes encoding the ketogenesis/lipid accumulation regulator 3-hydroxymethylglutaryl-CoA synthase 2 (*HMGCS2*), the lipo-inflammatory enzyme oleoyl-ACP-hydrolase *OLAH*, lipid metabolism-modifiers, such as the inner mitochondrial membrane protein transporter *SLC25A34* and the cartilage intermediate layer protein 2 (*CILP2*), the iron superoxide dismutase 1 (*FSD1*), or *MED12*, a subunit of the Mediator complex whose depletion promotes senescence-related cytokinesis failure and multinucleation [81].

**Table 1:** Top 20 up/down-regulated TVB-3116 TIS-specific DEGs.

Gene	Fold-change	*p*-value	Function
***SDR16C5***	6.147	1.54E-49	Retinol oxidation
***ST6GALNAC2***	5.468	8.28E-33	Sialic acid transference to glycoconjugates
*DNER*	5.278	1.70E-26	Insulin signaling/gluconeogenesis regulation
***PRUNE2***	5.207	6.95E-08	Post-endocytic trafficking
***MSMO1***	4.777	2.33E-50	Cholesterol biosynthesis
***S100P***	4.548	4.71E-61	Lipid metabolism rewiring/ferroptosis inhibition
*ACSS2*	4.543	2.61E-16	Acetyl-CoA synthesis (from acetate)
*ITIH6*	4.400	1.23E-09	Hyaluronic acid metabolism
***EPB41L3***	4.391	6.28E-07	Cytoskeletal protein-membrane anchor activity
***ABCG1***	4.256	3.44E-110	Phospholipids (sphingomyelin/cholesterol) efflux
*MVD*	4.097	5.79E-08	Cholesterol/sterol biosynthesis
***FDPS***	4.089	1.06E-07	Cholesterol/sterol biosynthesis
*STARD4*	3.981	1.82E-59	Intracellular transport of cholesterol
***PDP1***	3.782	1.16E-11	Acetyl-CoA synthesis (from pyruvate)
***INSIG1***	3.763	3.27E-39	Feedback control of cholesterol synthesis
*RUSC1-AS1*	3.685	6.45E-08	Vesicular trafficking
*ABCC8*	3.518	2.86E-04	Insulin secretion
***GPCPD1***	3.483	2.78E-55	Glycerophospholipid catabolism
*TMEM176A*	3.474	5.06E-12	Lipid metabolism
*TSPAN1*	3.428	1.90E-62	Suppression of NF-κB signaling
***ANGPTL4***	−26.911	4.52E-50	Triglycerides catabolism
***OLAH***	−18.576	3.73E-18	Release of free fatty acids from FASN
***SLC25A34***	−7.643	1.97E-10	Mitochondrial respiration and bioenergetics
***HMGCS2***	−7.145	7.82E-08	Ketogenesis/Acetyl-CoA catabolism
***CILP2***	−5.551	7.32E-36	Glucose metabolism
*PDK4*	−5.400	3.13E-09	Glucose to fatty acid energy switch
***FSD1***	−5.298	1.41E-10	Microtubule organization and stabilization
*P2RX5*	−4.751	7.24E-10	Purinergic pathway
*TMEM52B*	−4.304	2.64E-14	Extracellular exosome
*GAGE1*	−4.193	0.005	Cancer/testicular antigen
*ATP1A3*	−4.010	5.72E-50	Mitochondrial metabolic activity
*SLC16A1*	−3.940	1.39E-41	Monocarboxylate transporter
*BAG2*	−3.912	6.68E-11	Co-chaperone
*SLC19A3*	−3.740	7.19E-14	Thiamine metabolism
*SMIM3*	−3.716	0.012	PI3K/AKT regulator
***MED12L***	−3.715	6.08E-09	Mediator complex component
*EXTL1*	−3.664	0.003	Exostose family of glycosyltransferases
*TEX19*	−3.643	2.64E-09	Cancer/testicular antigen
*FABP6*	−3.643	7.07E-05	Lipid transport
*HSPG2*	−3.605	2.03E-38	Vascular extracellular matrix

*Bold* genes are shared with the ND-646 TIS-specific DEGs.

**Table 2:** Top 20 up/down-regulated ND-646 TIS-specific DEGs.

Gene	Fold-change	*p*-value	Function
***S100P***	9.463	4.73E-15	Lipid metabolism rewiring/ferroptosis inhibition
***ABCG1***	6.126	1.29E-64	Phospholipids (sphingomyelin/cholesterol) efflux
***PDP1***	5.005	4.32E-09	Acetyl-CoA synthesis (from pyruvate)
***SDR16C5***	4.719	1.89E-10	Retinol oxidation
*B4GALNT3*	4.477	1.76E-18	Galactosaminyltransferase
***GPCPD1***	4.411	1.24E-24	Glycerophospholipid catabolism
*SH3BGR*	4.240	4.34E-12	Cytoskeletal integrity
***MSMO1***	4.177	2.40E-12	Cholesterol biosynthesis
***FDPS***	4.122	1.91E-06	Cholesterol/sterol biosynthesis
*CD63*	3.995	1.41E-11	Lipid metabolism reprogramming
***PRUNE2***	3.873	4.01E-09	Post-endocytic trafficking
*ABHD3*	3.794	1.15E-22	Phospholipids remodeling
*ORAI3*	3.736	5.56E-14	Calcium signaling machinery
*VLDLR*	3.714	1.21E-16	Triglyceride/cholesterol metabolism
***ST6GALNAC2***	3.689	4.55E-08	Sialic acid transference to glycoconjugates
*RNF145*	3.663	2.18E-31	Lipid/cholesterol homeostasis
***EPB41L3***	3.552	1.76E-05	Cytoskeletal protein-membrane anchor activity
*BEND6*	3.551	5.15E-05	Transcription corepressor activity
*SCDP1*	3.534	4.71E-04	Cholesterol biosynthesis
***INSIG1***	3.514	3.95E-09	Feedback control of cholesterol synthesis
***HMGCS2***	−34.912	1.18E-30	Ketogenesis/Acetyl-CoA catabolism
***ANGPTL4***	−23.767	1.28E-40	Triglycerides catabolism
***OLAH***	−9.929	6.16E-09	Release of free fatty acids from FASN
***SLC25A34***	−8.953	3.95E-11	Mitochondrial respiration and bioenergetics
*MRVI1*	−7.113	2.22E-16	Calcium release
***CILP2***	−6.419	6.57E-32	Glucose metabolism
*KITLG*	−6.321	1.31E-41	Melanogenesis
*ACBD7*	−6.146	2.45E-26	Fatty-acyl-CoA binding activity
***FSD1***	−6.071	4.43E-11	Microtubule organization and stabilization
*DIO2*	−5.998	2.24E-23	Thyroid hormones biosynthesis
***MED12L***	−5.890	9.23E-12	Mediator complex component
*SNORD17*	−5.831	6.93E-06	Ribosomal RNA processing
*FANCB*	−5.463	4.48E-16	DNA repair
*HASPIN*	−5.218	3.59E-30	Chromosome and spindle function
*BRCA2*	−5.062	2.42E-11	DNA repair
*HELLS*	−5.009	1.28E-23	DNA strand separation
*STARD8*	−4.855	1.15E-05	Steroid hormone metabolism
*LRP2*	−4.810	6.28E-05	Steroid hormone metabolism
*SKIDA1*	−4.798	1.83E-13	DNA-binding transcription factor activity
*GRB14*	−4.752	8.49E-07	Insulin signaling

*Bold* genes are shared with the TVB-3166 TIS-specific DEGs.

To validate the RNA-seq-defined FASTIS signature independently, we performed quantitative RT-qPCR on a curated panel of transcripts representing the main functional programs altered by FASynis (Fig. 5B). This analysis confirmed statistically significant modulation of genes involved in cholesterol/lipid homeostasis and biosynthesis (e.g., *ABCG1*, *FDPS*, and *MSMO1*), acetate-derived acetyl-CoA production (*ACSS2*), and triglyceride/lipid turnover and membrane lipid remodeling (*ANGPTL4*, *OLAH*, *SCD*, *CD63*, and *PLSCR1*). It also confirmed modulation of numerous genes involved in immunodulatory and innate immune functions (e.g., *CCL22*, *CX3CL1*, *GDF15*, *IRF9*, *OAS1*, *OAS2*, and *LCN2*). Overall, the RT-qPCR data were directionally concordant with the RNA-seq results, strengthening the conclusion that the FASTIS state is associated with coordinated activation of metabolic and immune-related transcriptional remodeling rather than isolated gene-specific changes.

Functional enrichment analysis of the FASTIS DEGs that did not overlap with other TIS phenotypes using the EnrichR database revealed top-enriched GO terms, such as cholesterol homeostasis and mTORC1 signaling, while the only significantly depleted GO term was E2F target genes (Fig. 6B). Immunoblotting analyses revealed a significant reduction in phospho-p70 S6K1 in FASTIS cells, especially in the ND-646 FASTIS subtype. Since p70S6K is a canonical mTORC1 substrate, the dephosphorylation of p70S6K likely indicates metabolic stress-induced inhibition of downstream mTOR signaling rather than anabolic mTORC1 activation in FASTIS cells (Fig. 6B).

Taken together, these findings suggest that the transcriptional features unique to the FASTIS state involve an enrichment of genes involved in the biosynthesis, uptake, efflux, transport, storage, utilization, and/or excretion of lipids, particularly a significant shift towards pathways that produce cholesterol and acetyl-CoA, as well as the activation of immunomodulatory gene programs.

### The FASTIS state involves *on-target* engagement and a lipid-centered metabolic rewiring

**Fig. 7:**
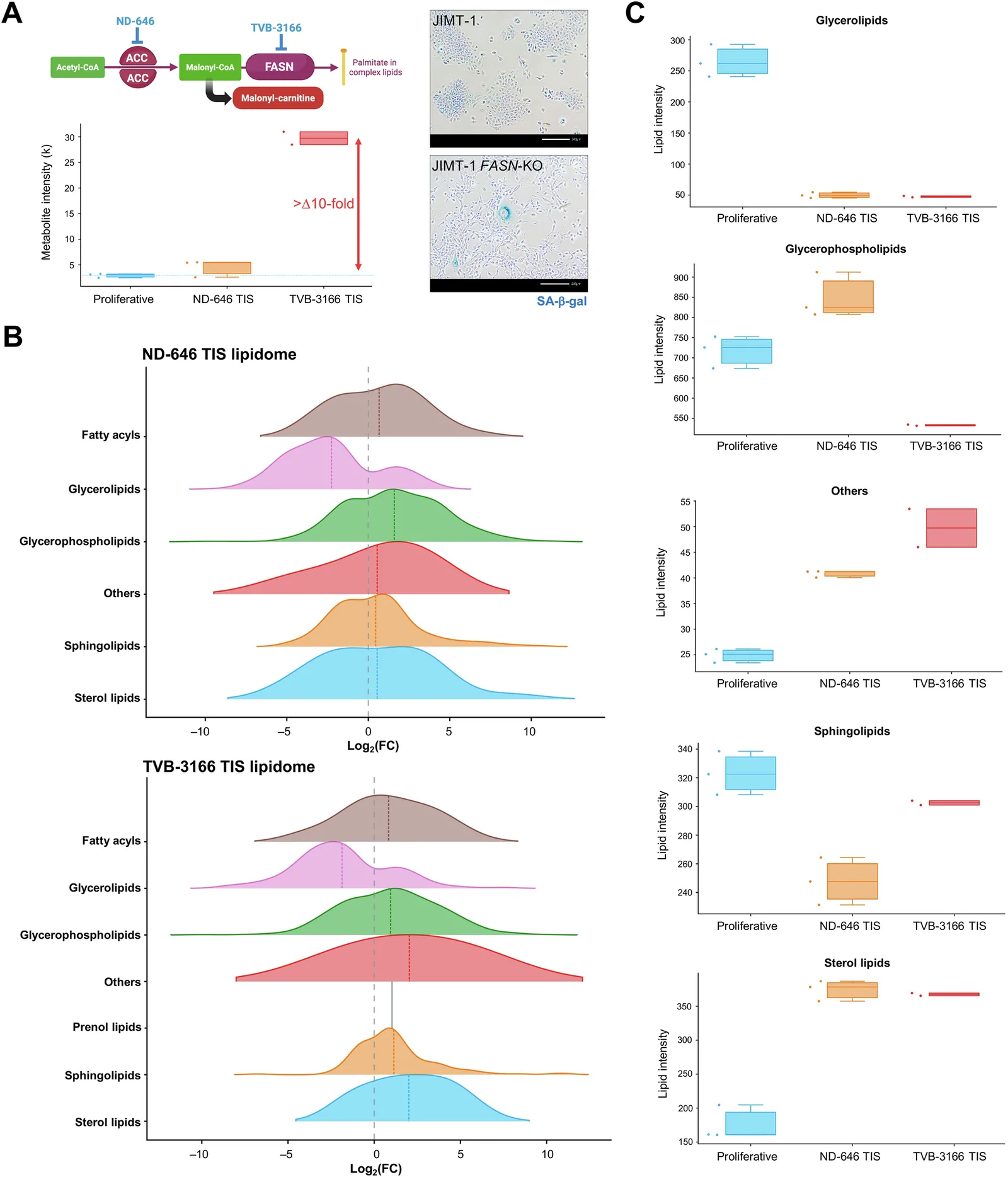
On-target lipogenesis blockade defines a glycerolipid-poor, sterol-rich FASTIS lipidome. **A** *Left*. Schematic representation of the de novo lipogenesis pathway indicating ACC and FASN as the targets of ND-646 and TVB-3166, respectively, and the accumulation of malonyl-L-carnitine as a surrogate of malonyl-CoA and a pharmacodynamic marker of FASN inhibition. The boxplot shows the intensity of the malonyl-L-carnitine metabolite in ND-646- and TVB-3166-induced TIS cells compared to proliferative controls. Representative SA-β-gal staining of parental JIMT-1 and JIMT-1 *FASN-KO* cells are shown on the right (scale bar: 200 μm). **B** Ridge-density plots showing the distribution of log2 fold change (FC) values of lipid-species in ND-646-induced TIS cells (*top*) and TVB-3166-induced TIS cells (*bottom*), each relative to proliferative cells. Lipid species are grouped by class: including fatty acyls, glycerolipids, glycerophospholipids, others, prenol lipids, sphingolipids, and sterol lipids. Vertical dashed lines indicate the class-specific central tendencies and the zero log_2_(FC) reference. **C** Box plots showing class-level lipid abundance for glycerolipids, glycerophospholipids, other lipids, sphingolipids, and sterol lipids in proliferative, ND-646-induced TIS and TVB-3166-induced TIS cells. Dots represent individual samples, and center lines indicate the median. Boxes indicate the interquartile range, and whiskers indicate the range (*n* = 3 for proliferative and ND-646 FASTIS cells and *n* = 2 for TVB-3166 FASTIS cells).

Before examining the broad metabolic architecture of FASTIS cells, we investigated whether our in vitro system reproduced a clinically validated pharmacodynamic metabolic hallmark of FASyn inhibition. In the first-in-human study of the TVB-3166 analog TVB-2640, serum malonyl-carnitine –a surrogate for the FASN substrate, malonyl-CoA– increased significantly with treatment, thereby providing evidence of *on-target* FASN engagement [44]. Conversely, ACC inhibition with ND-646 obstructs malonyl-CoA production, preventing malonyl-carnitine accumulation while inhibiting FASyn [41, 82, 83]. Consistent with this clinical benchmark, metabolomic profiling revealed a substantial (>10-fold) and specific accumulation of malonyl-L-carnitine upon acquisition of the FASTIS state in response to TVB-3166 but not in response to ND-646 compared to proliferative counterparts (Fig. 7A). Together with our confirmation of cancer cell refractoriness to TVB-3166 after *FASN* gene elimination in HAP1 cells by CRISPR/Cas9 [49] (Fig. S9), the spontaneous appearance of SA-β-gal^+^ cells *FASN-KO* JIMT-1 breast cancer cell cultures [84], and our inability to recover viable *FASN****-****KO* clones in the highly FASN-overexpressing SKBR3 cells, this metabolic pattern strongly supports the notion that FASTIS is a bona fide therapeutic outcome of *on target* engagement of FASyn inhibition.

Untargeted metabolome mapping revealed that the FASTIS phenotype comprises a chemically diverse but non-random metabolic landscape. ClassyFire annotation showed that, at the global level, the detected FASTIS-associated features were dominated by organic acids and derivatives, followed by lipids and lipid-like molecules, nucleosides and nucleotides, and organoheterocyclic compounds. In parallel, NPClassifier assigned the largest fraction of features to small peptides, along with additional representation of nucleosides, glycerophospholipids, fatty esters, fatty acids, and their conjugate derivatives (Fig. S10A).

When stratified by inducer, the FASTIS metabolomes by ND-646 and TVB-3166 showed both shared and distinct features. Using the ClassyFire annotation, ND-646 induced a more heterogeneous, bidirectional remodeling, with increased representation of the nucleoside/nucleotide, organic acid/derivative, organic acid, and organoheterocyclic classes. There was also marked depletion of lipid- and lipid-like- and organic-nitrogen features. NPClassifier further resolved this pattern as a decrease in fatty acids and conjugates, fatty esters, and glycerophospholipids, alongside an enrichment of small peptides, nucleosides, and related subclasses (Fig. S10B). Conversely, TVB-3166 generated a more restricted, coordinated metabolomic signature, which was dominated by decreases in the primary affected classes. These classes included lipids and lipid-like molecules, organic acids and derivatives and nucleoside-related metabolites, as annotated by ClassyFire. NPClassifier annotated the decreases as occurring in fatty acids/conjugates, glycerophospholipids, nucleosides, and small peptides.

Overall, these data suggest that FASTIS is associated with a prominent lipid-centered metabolic rewiring. The ACC inhibitor ND-646 elicits a broader bidirectional response, while the FASN inhibitor TVB-3166 imposes a more uniformly repressive metabolic program.

### FASTIS defines a glycerolipid-poor and sterol-rich lipidomic state with shared and ACC and FASN-specific lipid features

Having established that FASTIS is accompanied by broad metabolomic remodeling, we next asked whether this state is also associated with a distinct lipidomic architecture. PCA and PLS–DA both revealed a strong separation of proliferative cells from ND-646- and TVB-3166-induced FASTIS cells, with clear discrimination also between the two FASTIS-inducing agents (Fig. S11). This suggests that FASTIS is a biochemically distinct but class-specific lipidomic state. Further analysis of the chemical enrichment showed that the two FASTIS subtypes share glycerolipid depletion but differ in the lipid classes that accumulate preferentially (Fig. S11). ND-646-induced FASTIS exhibited a broader, more heterogeneous lipidomic shift, characterized by glycerophospholipid and sphingolipid enrichment, as well as additional changes in other lipid species. In contrast, TVB-3166-induced FASTIS exhibited a more focused, coordinated pattern, dominated by glycerophospholipid accumulation, along with concurrent increases in sphingolipids, fatty acyls and sterol lipids. Overall, these data identify the FASTIS state as a distinct lipidomic condition. They also suggest that such a condition converges on a shared glycerolipid-poor phenotype while reshaping membrane- and sterol-associated lipid pools in a differential manner depending on the inhibitor used (ACCi vs FASNi).

A more detailed analysis of the FASTIS lipidome revealed that both lipogenesis inhibitors share a core lipid remodeling program while retaining distinct inducer-specific characteristics. Ridge density analysis of log2 fold-changes showed that glycerolipids were the only lipid class consistently depleted in ND-646- and TVB-3166-induced FASTIS cells (Fig. 7B). This finding suggests that inducing depletion of this lipid pool is a common feature of the FASTIS state. In contrast, most of the remaining lipid classes showed distributions shifted toward positive values, indicating selective accumulation rather than global lipid collapse. In both conditions, fatty acyls and sterol lipids tended to become enriched, while the behavior of glycerophospholipids and sphingolipids depended more on the FASTIS-inducing agent (Fig. 7B). Overall, the crest profiles suggest that ND-646 produces a somewhat broader and more heterogeneous redistribution of lipid species, while TVB-3166 generates a more coordinated pattern centered on the depletion of glycerolipids and the accumulation of select non-glycerolipid classes.

The boxplot-based class summaries further clarified these shared and divergent trends (Fig. 7C). Glycerolipids decreased markedly in both FASTIS subtypes, dropping from the high levels observed in proliferative cells to similarly low levels in ND-646- and TVB-3166-driven FASTIS states. This defines the most robust common feature of the FASTIS lipidome. Sterol lipids increased significantly in both settings, reaching comparably high levels in ND-646- and TVB-3166-induced FASTIS cells (Fig. 7C). This finding reinforces the idea that sterol enrichment is another conserved hallmark of FASTIS. However, beyond these shared traits, the two lipidomes diverged. ND-646 FASTIS increased glycerophospholipids, whereas TVB-3166 FASTIS sharply reduced them. This revealed a major point of separation between ACC- and FASN-driven FASTIS states. Similarly, sphingolipids decreased in ND-646 FASTIS cells, but remained stable in TVB-3166 FASTIS cells. The composite “other” lipid fraction increased in both conditions, but more prominently in the latter. Overall, these results suggest that FASTIS is based on a common foundation of glycerolipid depletion and sterol accumulation. However, in response to ACC inhibition and FASN inhibition, FASTIS cells alter the surrounding lipid landscape differently. ND-646 promotes a more redistributive profile, while TVB-3166 induces a selective, polarized remodeling program.

**Fig. 8:**
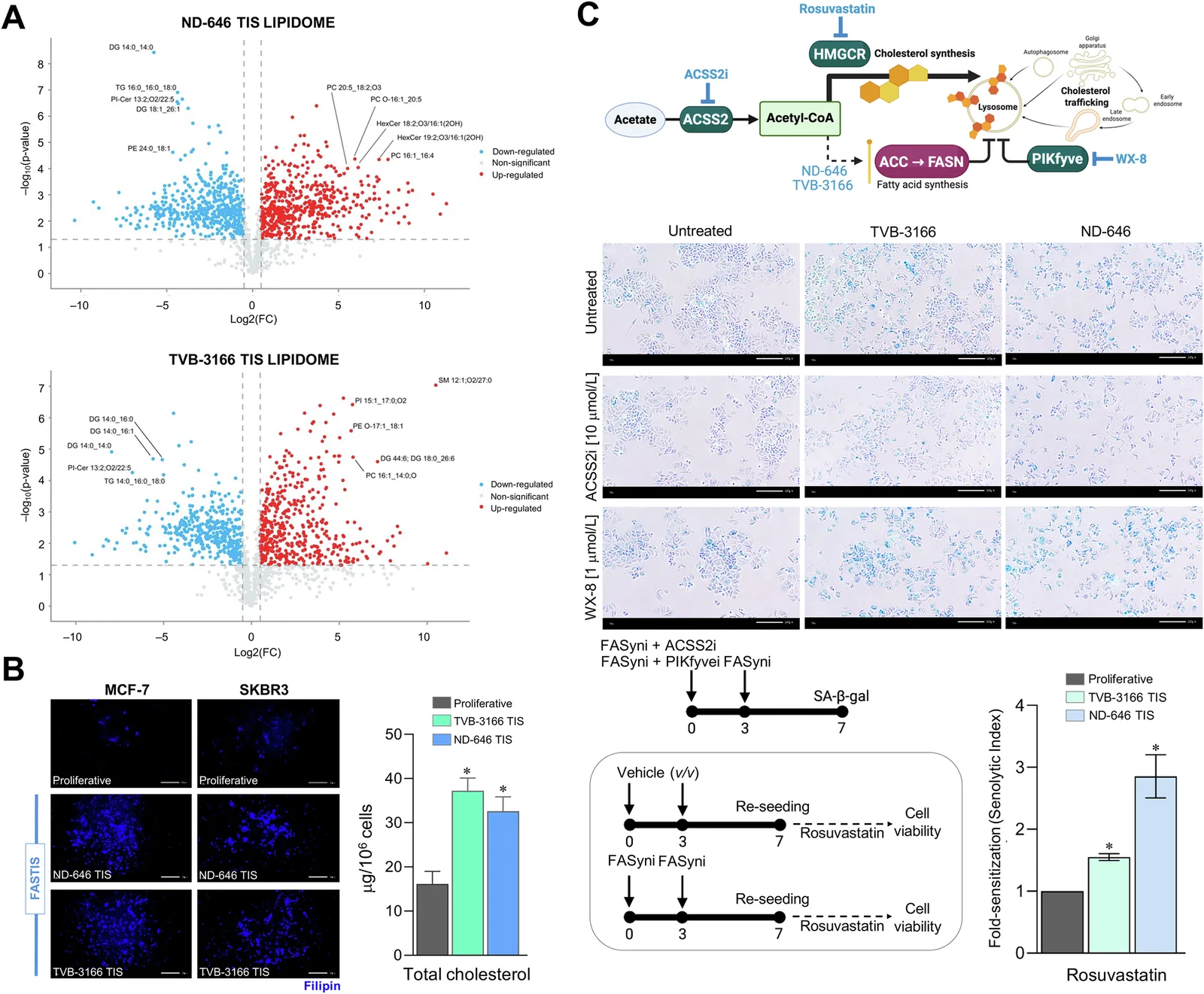
Lipidomic, cholesterol staining/quantification, and pharmacological analyses confirm the cholesterol-centric characteristics of FASTIS cells. **A** Volcano plots showing changes in lipid species in ND-646- and TVB-3166-induced TIS cells relative to proliferative controls. The *x*-axis shows log_2_(fold change), and the *y*-axis shows –log10(*p*- value). Individual lipid species are colored according to whether they are downregulated (blue), non-significant (gray), or upregulated (red), and selected annotated lipid species are indicated. **B** Representative filipin staining images of MCF-7 and SKBR3 cells under proliferative conditions and after FASTIS induction with ND-646 and TVB-3166. The bar plot shows quantification of total cholesterol (mean ± SD) for proliferative and TVB-3166- and ND-646-induced FASTIS cells. **C** A schematic of the acetate/acetyl-CoA, fatty acid synthesis, cholesterol synthesis, and cholesterol trafficking pathways, indicating the pharmacological interventions employed in the study: ACSS2i, ND-646, TVB-3166, rosuvastatin, and WX-8. Representative images of SA-β-staining (*n* = 3) are shown for untreated and TVB-3166- and ND-646-treated cells in the absence or presence of ACSS2i (10 μmol/L) or WX-8 (1 μmol/L). The treatment schedule used for ACSS2i/PIKfyvei co-treatment during FASTIS induction and subsequent SA-β-gal analysis at day 7 is shown below. A second scheme illustrates the workflow for the rosuvastatin challenge, including treatment with vehicle or FASyn inhibitors, re-seeding, and subsequent measurement of cell viability. The bar plot shows the fold-sensitization (senolytic index) to rosuvastatin in proliferative and TVB-3166-induced FASTIS and ND-646-induced FASTIS cells. Statistically significant differences between the proliferative and TIS phenotypes are shown (* *p* < 0.05) using ANOVA analysis.

At single-species resolution, volcano analysis identified a restricted set of high-confidence lipid changes that may serve as surrogate biomarkers of FASTIS, either shared by both lipogenesis inhibitors or preferentially associated with one subtype (Fig. 8A). A common signal emerging from both ND-646 and TVB-3166-induced FASTIS was the marked loss of selected neutral glycerolipid species, most notably DG 14:0_14:0 and TG 16:0_16:0_18:0, together with depletion of PI-Cer 13:2; O2/22:5. These concordant changes reinforce the idea that collapse of specific glycerolipids pools is a core lipidomic feature of the FASTIS state.

Beyond this common backbone, each inducer displayed a distinct lipid fingerprint (Fig. 8A). ND-646 FASTIS cells exhibited strong accumulation of specific hexosylceramides and phosphatidylcholine species, such as HexCer 18:2;O3/16:1(2OH) and HexCer 19:2;O3/16:1(2OH), as well as PC 16:1_16:4, and PC O-16:1_20:5. Meanwhile, lipids like PE 24:0_18:1 were reduced. Conversely, TVB-3166 FASTIS cells exhibited enrichment of a distinct set of lipids, including the sphingomyelin SM 12:1;O2/27:0, PI 15:1_17:0;O2, PE O-17:1_18:1, and highly unsaturated diacylglycerol species. This indicates a FASN-linked remodeling program with greater selectivity. Accompanying boxplot analyses confirmed the robustness of these changes and further emphasized that some lipid species behaved oppositely depending on the inhibitor (Fig. S12).

Taken together, these data highlight that FASTIS is built on both shared lipid hallmarks and drug-specific lipid signatures. Additionally, they identify a small set of lipids as potential surrogate biomarkers for distinguishing the common FASTIS state from its ACC- and FASN-driven subtypes.

### Cholesterol metabolism is mechanistically involved in generating and maintaining the FASTIS state

Due to the transcriptional enrichment of genes related to cholesterol homeostasis, such as mevalonate/cholesterogenic genes and cholesterol transport regulators, which suggests enhanced cholesterol biosynthesis, trafficking, and efflux, as well as the actual enrichment of sterol lipids in the lipidome of FASTIS cancer cells, we investigated the role of cholesterol in establishing the FASTIS fate using orthogonal approaches. To visualize intracellular cholesterol and sterol pools, we used the intrinsic fluorescence of filipin, a polyene macrolide that specifically binds to cholesterol and other sterols, serving as a reliable marker for their detection in cells [85, 86]. Filipin staining revealed higher cholesterol levels in ND-646- and TVB-3166-induced FASTIS cells than in their proliferative parental counterparts (Fig. 8B). Furthermore, we confirmed that intracellular cholesterol levels drastically increased by >2-fold in FASTIS cells using an ELISA-based cholesterol quantification assay (Fig. 8B). Together, these findings establish FASTIS as a bona fide cholesterogenic cell state involving a robust intracellular accumulation of cholesterol.

To demonstrate the role of cholesterol metabolism in generating and maintaining the FASTIS state, we pharmacologically examined the cholesterol axis at three levels (Fig. 8C). First, we restricted the upstream supply of acetate-derived acetyl-CoA to the cholesterogenic branch using an ACSS2 inhibitor [87, 88]. Second, we disrupted the handling of intracellular cholesterol in the lysosomal/endolysosomal compartment using an inhibitor of the lipid kinase PIKfyve [89–91]. Third, we used an HMG-CoA reductase (HMGCR) inhibitor to block the downstream mevalonate step required for the de novo cholesterol synthesis. Examining the emergence of SA-β-gal^+^ FASTIS cells with the ACSS2 inhibitor ACSS2i co-added during the first three days of ND-646 and TVB-3166 treatment revealed a reduction in cells acquiring an SA-β-gal^+^ FASTIS state (Fig. 8C). Conversely, blocking cholesterol exit from lysosomal membranes with WX-8 concurrently during the first three days of ND-646 and TVB-3166 treatment resulted in a significant increase in SA-β-gal^+^ FASTIS cells and a significant expansion of the SA-lysosomal compartment (Fig. 8C, S13). Finally, we observed a significant increase (up to ∼3-fold) in the FASTIS cells’ responsiveness to the HMGCR inhibitor rosuvastatin compared to their proliferative counterparts (Fig. 8C), thereby revealing a “FASTIS-lytic” activity of statins. Taken together, these findings support the notion that the FASTIS fate depends on an active acetyl-CoA supply, endolysosomal cholesterol trafficking/storage, and cholesterol biosynthetic flux.

### The FASTIS-associated secretory phenotype (FASASP) has a low inflammatory but immunogenic profile

**Fig. 9:**
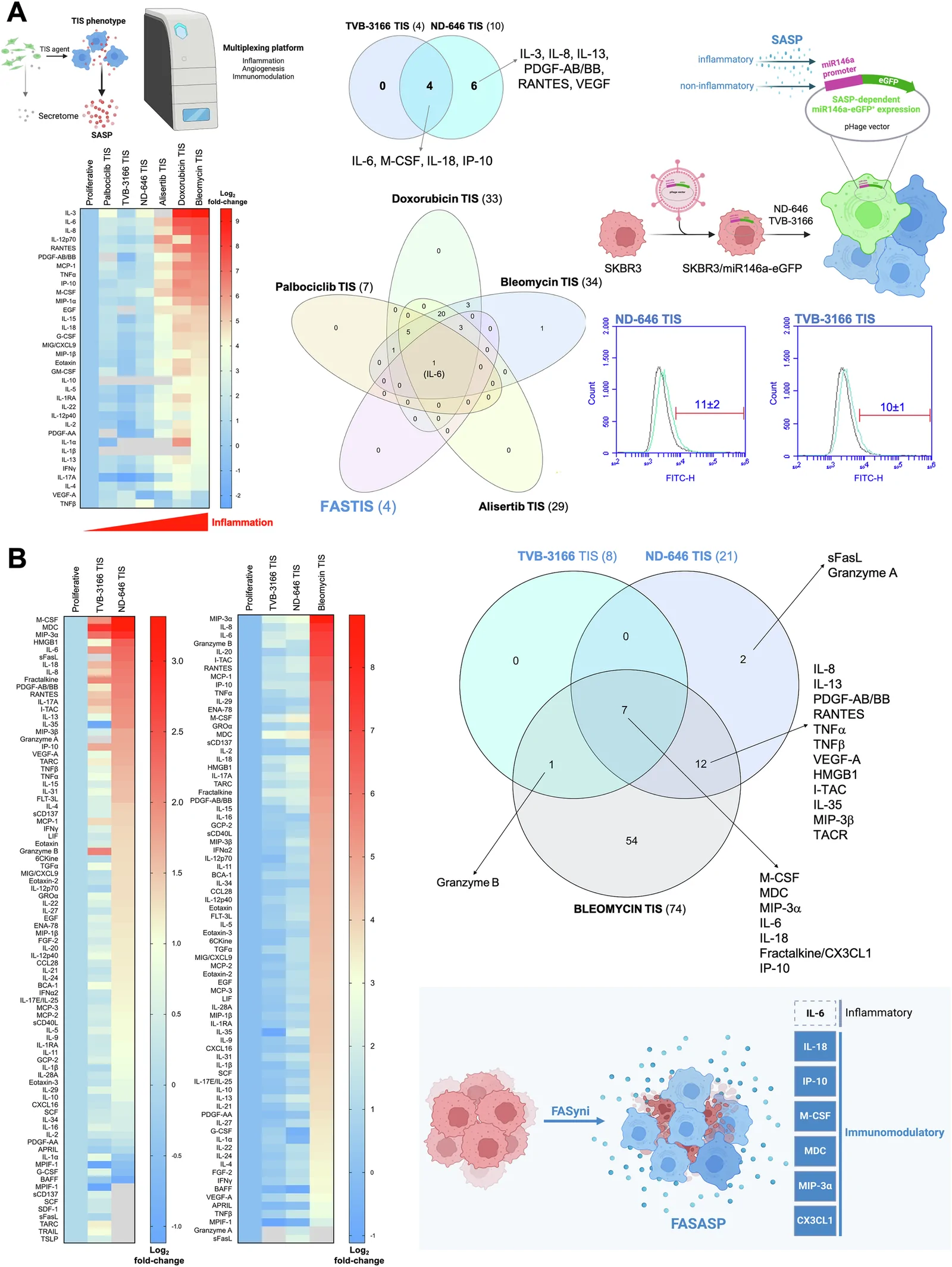
The FASTIS-associated secretome is enriched for immunomodulatory factors. **A** *Left*. The qualitative and quantitative composition of the FASTIS-associated secretome was characterized using multiplex bead-based immunoassays. Heatmap showing the log2 fold-change values of 34 cytokine and chemokine biomarkers (Human Cyto/Chemo/GF Panel A 38 Plex Kit) in the cell supernatant of untreated (proliferative) and TIS SKBR3 cells (7 days). The red and blue colors in the heat map represent higher and lower log2 fold-change values, respectively*. Middle*. Venn diagrams showing unique and common secretome components for either the TVB-3166 *vs*. ND-646 TIS comparisons or for all of the TIS secretome comparisons using an arbitrary TIS/proliferative ratio cut-off of log2FC ≥ 1.58 (above 3-fold enrichment). *Right*. Activation levels of the negative feedback regulator of the SASP response miRNA-146a in FASTIS SKBR3 breast cancer cells. The inflammatory SASP-responsive/NF-κB-driven miR146a-eGFP reporter was transduced into SKBR3 cells, FASTIS was induced by treatment with ND-646 and TVB-3166 and eGFP fluorescence was measured by flow cytometry. Data are mean fluorescence intensities (arbitrary units ±S.D.) from three independent experiments. **B** *Left*. Heatmap showing the log2 fold-change values of 96 cytokines, chemokines, and growth factors (Cytokine/Chemokine 96-Plex Discovery Assay^®^ Array, HD96) in the cell supernatant of untreated (proliferative) and TIS SKBR3 cells (7 days). The red and blue colors in the heat map represent higher and lower log2 fold-change values, respectively. *Right*. Venn diagrams showing unique and common secretome components for the TVB-3166 TIS-, ND-646 TIS-, and bleomycin TIS-associated secretome comparisons using an arbitrary TIS/proliferative ratio cut-off of log2FC ≥ 1.58 (above 3-fold enrichment). The FASTIS-associated secretome (FASASP) is highly enriched for immunomodulatory (6 out of 7) vs. inflammatory (1 out of 7) factors.

The composition and strength of the SASP or “senescence-messaging secretome” that mediates the (patho)physiological effects of senescent cells may vary substantially depending not only on the duration of senescence, the environment and cell type, but also on the senescence-inducing mechanism [2–4, 17, 92, 93]. To investigate whether the SASP generated by the FASTIS phenotype is similar/dissimilar to that of other TIS inducers, we used the Luminex^®^ platform to perform cytokine arrays with conditioned media from proliferative and TIS SKBR3 cells (Fig. 9A, *left*). When we performed a categorization based on the ratio of cytokine, chemokine, and growth factor abundance between the TIS phenotypes and their replicative control counterparts, we found that the secretory profiles were TIS inducer-dependent both in the number of SASP components and in their abundance. We observed two different sets of inflammatory profiles, a high inflammatory profile including the SASPs of bleomycin, doxorubicin, and alisertib TIS and a low inflammatory profile including the SASPs of ND-646, TVB-3166 and palbociclib TIS (Fig. 9A, *left*). A TIS/proliferative ratio cut-off of log2FC ≥ 1.58 (above 3-fold enrichment) was then arbitrarily assigned to differentiate the FASTIS-associated SASP components (IL-6, M-CSF, IL-18, IP-10) and their overlapping/specificity compared to other TIS phenotypes. While most of the SASP components were found to be commonly highly secreted among the inflammatory TIS phenotypes (bleomycin, doxorubicin, and alisertib), only a few of them were shared by low-inflammatory TIS phenotypes (ND-646, TVB-3166, and palbociclib).

To confirm further the low inflammatory nature of the FASTIS-associated SASP, we took advantage of the microRNA 146a (miR146a), a miRNA commonly known for its antagonistic role in inflammation and for its dampening of immune responses. miR146a is expressed in response to elevated inflammatory cytokine levels as part of a negative feedback loop that restrains excessive SASP activity to limit the deleterious effects of the high levels of SASP factors on surrounding tissues [18, 25, 94, 95]. We engineered ACC/FASN-overexpressing SKBR3 cells to express a construct containing a SASP reporter construct with the miR146a promoter fused to eGFP (SKBR3/miR146a-eGFP cells) as a surrogate marker of miR146a induction following treatment of cells with the panel of senescence inducers (Fig. 9A, *right*). The ability of the FASTIS-associated SASP to activate miR146a was dramatically poor, as flow cytometry analyses confirmed that the percentage of miR146a-positive cells was as low as 10% in ND-646- and TVB-3166-induced senescent cells (Fig. 9A, *right*). Since upregulation of miR146a reflects the severity of SASP inflammation, such that cells undergoing senescence without the induction of a robust inflammatory SASP do not express miR146a, our results confirm the low inflammatory profile of FASASP. We then reanalyzed the conditioned media from ND-646/TVB-3166 TIS and bleomycin TIS cells using a multiplex panel more enriched for immunomodulatory factors. Interestingly, the extracellular milieu of ND-646/TVB-3166 TIS cells maintained the expression of several immunomodulatory factors, some of which were specific and expressed at high intensity in the FASTIS phenotype (IL-18, IP-10, M-CSF, MDC, MIP-3α, CX3CL1; Fig. 9B).

Unlike the pro-inflammatory SASP associated with DNA-damaging TIS agents, the FASASP (FASTIS-associated SASP) is a less inflammatory secretome enriched with immunodulatory factors (Fig. 9B).

### FASTIS cells exhibit an increased overall priming for mitochondrial apoptosis

**Fig. 10:**
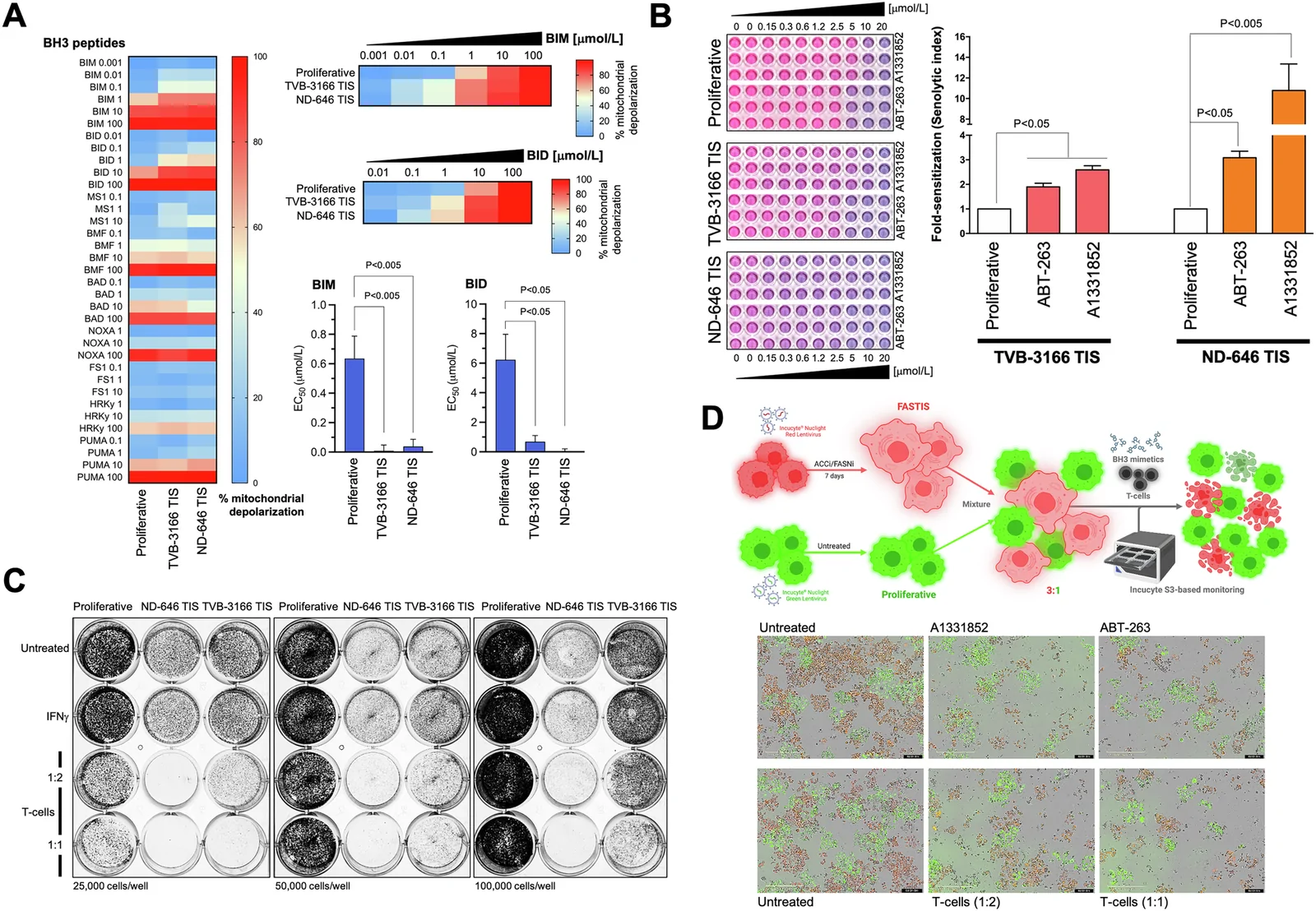
FASTIS breast cancer cells acquire hypersensitivity to BCL-xL-targeting BH3 mimetics and T cell-based immunotherapy. **A** Plate-based JC-1 BH3 profiling was performed to measure mitochondrial depolarization in proliferative (untreated), TVB-3166 TIS, and ND-646 TIS SKBR3 cancer cells exposed to activator (BIM, BID, PUMA) and sensitizer (BMF, BAD, NOXA, HRK) BH3 peptides. Heatmaps show % mitochondrial depolarization caused by increasing concentrations of activator and sensitizer peptides. Data shown are the mean of ≥3 independent experiments using three technical replicates for each peptide. Graphs represent the means (*columns*) ± S.E.M. (*bars*) of ≥3 independent experiments BIM and BID EC_50s_ (μmol/L) in proliferative and FASTIS SKBR3 breast cancer cells. Statistically significant differences *(*ANOVA*)* between the proliferative and FASTIS phenotypes are shown. **B** Proliferative (untreated) and FASTIS SKBR3 breast cancer cells were replated into 96-well plates (8000 cells/well) and cultured with increasing concentrations of the BH3 mimetics ABT-199/venetoclax and A1331852. After 5 days, cell viability was measured using the alarmarBlue™ assay. Shown are representative images of alamarBlue™-based cell viability assays showing changes in the number of metabolically active cells in response to serial dilutions of BH3 mimetics. Bar graphs of the senolytic indexes obtained by dividing the IC_50_ values of ABT-263/navitoclax and A1331852 in proliferative SKBR3 cells by those obtained in FASTIS derivatives. Data represent the mean ± S.D. of ≥3 independent experiments performed in triplicate. Statistically significant differences (ANOVA analysis) between proliferative and TIS phenotypes means are shown. **C** Increasing numbers (25,000 cells/well, 50,000 cells/well or 100,000 cells/well) of proliferative (untreated) and FASTIS SKBR3 breast cancer cells were challenged either with IFNγ or cytokine-activated T (CAT) cells at 0.5:1 and 1:1 E (CAT cells): T (breast cancer cells) ratios. Tumor cells were fixed and stained, and subjected to crystal violet staining 2 days later. Representative microphotographs (*n* = 3) are shown. **D** Mixed cultures of nuclear-labeled versions of proliferative (green; 2000 cells/well) and FASTIS (red; 6000 cells/well) SKBR3 cells stably generated by transduction with Incucyte^®^ Nuclight (red or green) lentiviruses were plated in 96-well plates and treated with ABT-263/navitoclax (1 μmol/L), A1331852 (1 μmol/L) or cytokine-activated T (CAT) cells at 0.5:1 and 1:1 E (CAT cells): T (breast cancer cells) ratios. The change in red and green nucleus cell populations before and after treatments was monitored for 6 days using the IncuCyte^®^ S3 Live-Cell Analysis System.

A paradoxical feature of senescent cells is that they are refractory to most apoptosis-inducing therapeutics but variably sensitive to apoptosis-inducing BH3 senolytics, which often involves a reorganization of the balance between pro-apoptotic and pro-survival BCL2 proteins [22–26, 70, 73, 96]. To gain insight into the dynamic changes of the BCL-2:BH3 interactome that might underlie the senescence versus death fate determination that occurs in the FASTIS state, we measured the relative levels of mitochondrial priming in FASTIS cancer cells using the so-called BH3 profiling method [22–26, 73, 96] (Fig. 10A). The BH3 profiling is a functional tool that assesses mitochondrial susceptibility to mitochondrial outer membrane permeabilization (MOMP) in response to synthetic peptides encoding the active domains of pro-apoptotic BH3 proteins (BIM, BID, BAD, PUMA, NOXA, MS1, FS1, BMF, HRK) that interact promiscuously or selectively with pro-survival BCL-2 family proteins [97–99]. The BH3 profiling assay can therefore determine both the state of overall mitochondrial priming –also known as the “readiness” to undergo apoptosis– and map the specific dependence on one or more anti-apoptotic (BCL-2, BCL-xL, BCL-w, MCL-1, BFL-1) proteins, also known as “apoptotic blockade”.

Peptide titration experiments revealed that the extent of depolarization solely differed significantly between proliferative and FASTIS cells when using activator (particularly BIM and BID) but not sensitizing (BAD, NOXA, HRK, and BMF) BH3 peptides. The 50% effective concentration (EC_50_) values for BIM and BID were notably lower in the FASTIS phenotypes compared to the proliferative counterparts. Thus, ∼15 to ∼70 lower amounts of the BIM peptide were necessary to induce MOMP in TVB-3166- and ND646-induced senescent cells, respectively, compared to proliferative (untreated) cells (Fig. 10A). Similarly, ∼10 to ∼200 lower amounts of the BID peptide were sufficient to induce MOMP in TVB-3166- and ND646-induced senescent cells, respectively, compared to the untreated proliferative controls (Fig. 10A). As BIM or BID peptides can bind to any of the antiapoptotic proteins and can also directly bind and activate BAX and/or BAK, they can be used to determine the overall level of mitochondrial priming because the dose required to induce MOMP is inversely correlated with the level of mitochondrial priming (e.g., a low dose of BIM/BID required indicates that the cell has a high level of priming and *viceversa*). Our results, therefore, suggest that FASTIS breast cancer cells, compared to their proliferative parental cells, have a higher overall level of mitochondrial priming –the proximity to the apoptotic threshold– in the absence of overt apoptotic blockade or dependence on a specific antiapoptotic protein.

### FASTIS cells are hypersensitive to BH3 mimetics and T cell-based immunotherapy

BCL-2 family-targeted BH3 mimetics, particularly those targeting BCL-xL, can redirect senescent cells toward apoptosis [24–27]. The mitochondrial priming status also affects cancer-cell susceptibility to NK/T-cells-mediated killing [100]. The above BH3 profiling results showed that mitochondria in FASTIS cancer cells have a small reserve of unbound pro-survival proteins (e.g., BCL-xL) as they are rapidly and completely depolarized by low doses of the promiscuous BH3-only activators BIM/BID. In addition, since selective sensitizing peptides had little or no effect, FASTIS cancer cells do not appear to exhibit a specific apoptotic blockade dependent on the overexpression of a specific anti-apoptotic protein. We therefore hypothesized that BCL-xL-targeting BH3 mimetics and T cell-based immunotherapy might push FASTIS breast cancer cells across the apoptotic threshold, ultimately leading to their (immuno)senolysis.

We first performed cell viability bioassays in proliferative SKBR3 cells and FASTIS derivatives using alamarBlue*™*, which contains an oxidation-reduction (REDOX) indicator (resazurin) that both fluoresces and changes color in response to the metabolic activity of viable cells. Dose-response assays to graded concentrations of the BCL-2/BCL-X_L_ inhibitor ABT-263/navitoclax revealed senolytic indexes (IC_50 proliferative_/IC_50 FASTIS_) of ∼2 in TVB-3166 TIS cells and ∼3 in ND-646 TIS cells (Fig. 10B). The FASTIS phenotype was exquisitely sensitive to the BCL-X_L_-specific inhibitor A1331852, as senolytic indexes as high as ∼10 were observed in ND-646 TIS cells (Fig. 10B). To test the hypothesis that primed FASTIS cancer cells may be more sensitive to immunocytolysis, we exposed them to cytokine-activated T cells (CATs) cells, a form of cell-based immunotherapy consisting of a heterogeneous population of effector T cells generated from peripheral blood mononuclear cells (PBMCs) cultured under cytokine stimulation, and possessing non-MHC-restricted cytolytic activity against tumor cells [84, 101, 102]. Plaque formation assays using crystal violet staining revealed an increased responsiveness of FASTIS cancer cells, especially ND-646 TIS breast cancer cells, to increasing ratios of CATs (Fig. 10C).

We finally generated clonal nuclear-labeled SKBR3 cells by lentiviral transduction and antibiotic selection (Fig. 10D, *top*). We plated mixed cultures of proliferating (green nuclei) and FASTIS (red nuclei) SKBR3 cells (1:3 ratio) and one day later we added graded concentrations of BH3 mimetics or different ratios of CATs to cancer cells (1:2 and 1:1). When cells were monitored for cell lysis by fluorescence microscopy using the IncuCyte S3 live-cell analysis system, the lytic effect of BH3 mimetics and CATs was largely restricted to red nucleus-labeled FASTIS cells while green nucleus-labeled cells remained freely proliferating (Fig. 10D, *bottom*), further demonstrating that FASTIS cancer cells are easier targets for CATs.

Taken together, these results suggest that the FASTIS state increases the susceptibility of cancer cells to senolysis by BCL-X_L_-targeting BH3 mimetics and T-cell-based immunotherapy.

## Discussion

**Fig. 11:**
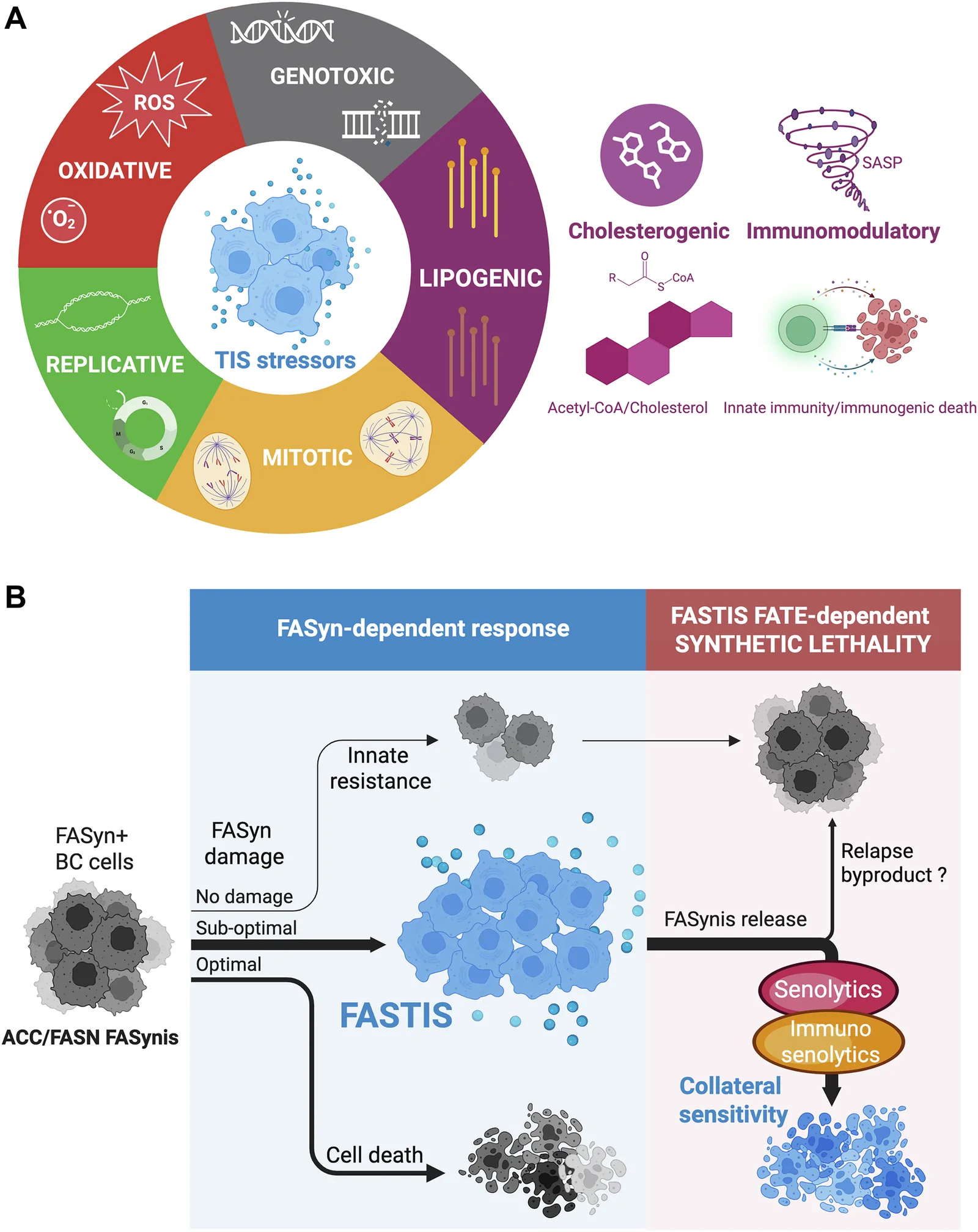
FASTIS: a novel TIS phenotype with therapeutic implications. **A** Lipogenic stress induced by the pharmacological blockade of FASyn drives a new type of TIS characterized by profound metabolic changes, including the activation of pathways the produce and handle intracellular cholesterol and acetyl-CoA, as well as the activation of innate immune-like responses in cancer cells. Consequently, immunomodulatory factors are overrepresented in the secretome associated with the FASTIS phenotype (FASASP), which is largely devoid of inflammatory components. **B** This study provides evidence for a two-step “one-two punch” approach that could enable the rational exploitation of synthetic lethality due to lipogenic stress-related senescence. First, the model predicts that ACCi and FASNi therapies can potently inhibit lipogenesis-dependent breast cancer proliferation, yet they are insufficient to promote extensive and robust apoptotic cell death. Second, FASTIS is the favored metastable state (the thicker lines represent more likely events) of lipogenesis-addicted breast cancer cells exposed to FASynis, enabling an acquired susceptible to the senolytic activity of statins, BCL-xL-targeting BH3 mimetics, and T-cell-based immunotherapies. These synthetic lethal vulnerabilities of the FASTIS state therefore result from a cell fate decision to escape cell death and enter survival mode in a cellular senescence state with targetable molecular and metabolic drivers. Companion diagnostics, theragnostic biomarkers, and new families of FASTIS-targeted senotherapeutics can be developed based on the comprehensive characterization of the druggable molecular and metabolic liabilities of FASTIS cells. A FASTIS-based “one-two punch” senogenic-(immuno)senolytic approach would maximize the clinical benefit of FASynis and, as treatments are administered sequentially, this strategy is also expected to reduce the toxicity associated with combination therapies.

TIS can occur when cancer cells are exposed to therapeutics that induce oxidative, mitotic, genotoxic, or replicative stress. Here, we add targeted inhibition of the core enzymes in the de novo lipogenesis pathway (ACC and FASN) to the list of cellular stresses that can induce a TIS state in tumor cells (Fig. 11A). We term this phenomenon FASTIS (Fatty Acid Synthesis Therapy-Induced Senescence). Systematically deconstructing the molecular and metabolic features of FASTIS allowed us to identify a unique FASTIS-encoding transcriptomic signature involving lipogenic rewiring and activation of cell-intrinsic innate immune responses including an increased sensitivity to immune checkpoint induction, the acquisition of a cholesterol-enriched lipidome that is necessary for the generation and maintenance of the FASTIS state, the development of a distinct FASTIS-associated immunomodulatory but poorly inflammatory secretome (FASASP), and the occurrence of FASTIS-associated vulnerabilities that can be exploited therapeutically with acetyl-CoA/cholesterol metabolism-targeting drugs, BCL-xL-targeting BH3 senolytics, and T cell-based immunotherapy (Fig. 11B).

The cytotoxic responses of cancer cells to the pharmacological blockade of endogenous de novo FASyn with ND-646 and TVB-3166 mirror basal ACC and FASN expression: the higher these proteins are expressed, the more dependent the cancer cells are on FASyn for survival. However, although ACCis and FASNis efficiently suppress proliferation in FASyn-dependent cells, they induce little apoptotic death. This is true even in HER2 + SKBR3 breast cancer cells, which have the highest reported FASN content of any established human cell line –up to 28% of cytosolic protein weight [46]. Therefore, we speculated that lipogenic stress induced by FASyn inhibition may trigger a senescence program that preserves cell survival in cells that fail to undergo apoptosis. Consistent with this idea, prolonged exposure to clinically relevant FASynis drives ACC/FASN-overexpressing breast cancer cells into a senescent-like state. This state is marked by cytomorphological remodeling, increased SA-β-gal activity, and strong upregulation of cell cycle inhibitors, especially the senescence-initiating driver p21^WAF1/CIP1^. Because ACC/FASN-overexpressing SKBR3 cells are *TP53* mutant, the increase in p21^WAF1/CIP1^ was unexpected. However, *TP53*-independent activation of the Chk2-p21^WAF1/CIP1^ DNA damage response (DDR) axis has been associated with transient TIS following exposure to the PPAR inhibitor olaparib [70]. Additionally, p53-independent activation of p21^WAF1/CIP1^ has been observed in contexts of embryonic, oncogenic, metabolic, and hypoxia-associated senescence [103–105]. Importantly, FASTIS does not produce permanent arrest in *p53*-deficient SKBR3 cells, and clonogenic capacity is partially restored after drug withdrawal. The metastable nature seems to require sustained FASyni exposure; pulsed treatment increased SA-β-gal-positive cells, particularly at moderate doses. Similar to the CDK4/6 inhibitor palbociclib, these results suggest that FASTIS maintenance requires continuous signaling and intact p53 function for a deeper senescence response in cancer cells. FASTIS cells that re-enter the cell-cycle may represent a post-senescent state with distinct functional and clinically relevant implications that should be explored [71, 106]. Since p57^Kip2^ and p27^Kip1^ can also delay cell cycle progression to support stress survival [107], future work should determine whether transient DDR pathways, canonical or non-canonical, help establish FASTIS under oxidative or replicative stress without direct DNA damage.

Transcriptomic profiling revealed that FASTIS exhibits gene expression and enrichment patterns similar to those of other TIS states induced by bleomycin, alisertib, doxorubicin, and palbociclib, while displaying unique characteristics. On the one hand, FASTIS exhibited a strong association with a core senescence transcriptional program. Both ND-646- and TVB-3166-induced TIS scored ≥0.95 with SENCAN, a machine learning–based senescence predictor that was originally trained using 13 cancer cell lines from four different cancer types that were treated with alisertib or etoposide [73]. SENCAN is built on 137 genes, including not only canonical senescence markers (e.g., *CDKN1A*, *HMGB1*) but also genes involved in mRNA processing/splicing, interferon signaling, and transcriptional readthrough. This makes it particularly well-suited for detecting senescence in cancer cells compared with conventional signatures derived from normal cells. Notably, the FASTIS transcriptional profile includes double-stranded (ds) RNA-sensing factors and interferon-inducible antiviral genes (e.g., *OAS1*, *OAS2, IRF9*), which have recently been proposed as markers of TIS cancer cells [72]. GSEA of both FASTIS-inducing treatments revealed shared positive enrichment for cholesterol homeostasis and TNFα/interferon (α/γ)-related immunomodulatory responses. These responses include genes linked to stress-activated lipogenesis (*SCD*, *ACSS2*), membrane remodeling (*PLSCR1*), restraint of proinflammatory cytokine production (*PHLDA1*), and anti-tumor immunity (*TNFSF15*, *CX3CL1/fractalkine*). Both FASTIS transcriptomes exhibited negative enrichment for E2F targets, the G_2_/M checkpoint, MYC targets, and mitotic spindle hallmarks. Consistently, functional analysis highlighted the strong involvement of the mitotic cell-cycle, protein binding, and spindle/chromosome segregation processes. On the other hand, genes that were uniquely altered in FASTIS and not in other TIS states again pointed to increased cholesterol homeostasis and the marked depletion of E2F targets. Thus, FASTIS appears to activate cholesterogenic and alternative stress-lipogenic pathways to preserve acetyl-CoA availability by inhibiting EF2-controlled programs necessary for DNA replication, chromosome segregation, and cytokinesis. This finding suggests a potential mechanistic connection between endogenous lipogenesis and mitotic fidelity, particularly given that de novo lipids are directed to the nuclear envelope during mitosis [108]. Although mTORC1-related gene sets showed mixed enrichment, FASTIS cells displayed pronounced p70S6K dephosphorylation. This is consistent with reduced mTORC1 activity and suppressed protein translation, supporting the idea that diminished protein synthesis contributes directly to senescence-associated cell-cycle withdrawal [72].

Our metabolomic and lipidomic data reveal that the FASTIS state is not simply characterized by the collapse of lipid anabolism. Instead, ACC and FASN blockade led to a consistent glycerolipid-poor, sterol-rich state with significant intracellular cholesterol accumulation. Clear divergence occurred only in the surrounding glycerophospholipid and sphingolipid landscapes. This important distinction suggests that FASTIS is driven less by the depletion of bulk lipids and more by a failure to maintain the selective membrane lipid architectures necessary for organelle homeostasis. One parsimonious model is that, following ACC/FASN inhibition, not only is cholesterol synthesized or retained in excess, but it is also repartitioned aberrantly to the endolysosomal compartment. This idea aligns with our observations that FASTIS is accompanied by rewired cholesterol homeostasis and trafficking transcriptional programs, that intracellular cholesterol drastically rises in FASTIS cells, that ACSS2 inhibition suppresses FASTIS entry, and that promoting lysosomal cholesterol retention with a PIKfyve-directed perturbation intensifies the FASTIS phenotype. It also aligns with recent findings indicating that senescent cells accumulate cholesterol in lysosomes by rerouting cholesterol flux machinery [109–111]. This generates cholesterol-rich lysosomal microdomains that sustain senescence-associated signaling. The lysosomal model provides a mechanistic bridge between FASyn blockade and the exaggerated senescent phenotype observed when cholesterol handling is further disrupted. De novo lipogenesis has been shown to sustain the phosphatidylinositol species and the membrane properties necessary for lysosome maturation, autophagosome-lysosome fusion and membrane recycling [112]. Loss of FASN alters phosphoinositide levels, changes acyl-chain composition, and compromises lysosomal function. One might be tempted to suggest that ACC/FASN inhibition creates a membrane state that allows for impaired cholesterol egress from lysosomes. In contrast, PIKfyve inhibition exacerbates this bottleneck by convergingly acting on the same phosphoinositide-dependent trafficking axis. According to this perspective, lysosomal cholesterol accumulation in the FASTIS state is not merely a secondary byproduct of FASyn inhibition, but rather a part of the machinery that stabilizes it.

ACSS2 may provide an additional level of control over the FASTIS fate. ACSS2 is ideally positioned to maintain sterol output when palmitate synthesis is blocked because it supplies acetyl-CoA derived from acetate [87]. Since acetyl-CoA regulates major downstream metabolic pathways of fatty acid and cholesterol synthesis, the observed upregulation of ACSS2 and the downregulation of HMGCS2, along with the activation of cholesterol homeostasis-related genes and the significant accumulation of intracellular cholesterol, are consistent with an adaptive, lipid-supporting pathway being activated in FASTIS cells. In this pathway, FASTIS cells might redirect acetyl-CoA away from de novo lipogenesis and ketogenesis and toward cholesterol biosynthesis. Accordingly, ACSS2 inhibition limits the acquisition of the SA-β-gal^+^ FASTIS phenotype. However, ACSS2 may have functions beyond supporting cholesterol synthesis. Recent studies show that ACSS2 sustains senescence programs by channeling local acetyl-CoA into the acetylation-dependent regulation of SASP competence [113, 114]. Therefore, FASTIS cells may exploit ACSS2 in two ways: to preserve the flux of acetyl-CoA into the mevalonate/cholesterol branch and to reinforce the senescent state by regulating its secretory program through acetylation at the chromatin level. This framework also explains the statin result. If cholesterol biosynthesis helps “build” the FASTIS phenotype and established FASTIS cells become increasingly dependent on that pathway, then HMGCR inhibition should acquire a FASTIS-lytic function rather than behaving only as an upstream metabolic blocker. Our observation that FASTIS cells become markedly more sensitive to rosuvastatin is fully consistent with this idea. More broadly, these findings suggest that lysosomal cholesterol partitioning, phosphoinositide-dependent membrane trafficking, and ACSS2-driven acetyl-CoA flux may work together as a single adaptive circuit engaged by cancer cells upon FASyn blockade. Thus, FASTIS is best viewed as a cholesterol-dependent senescent state that creates its own therapeutic liabilities rather than as a passive endpoint of lipogenic stress.

We acknowledge that FASN activity has been shown to support the early establishment of senescence in normal fibroblasts and hepatic stellate cells. FASN appears to function as a metabolic enabler that sustains the mitochondrial bioenergetic requirements of the senescence program, including features associated with the secretory phenotype (SASP) [115]. Inhibiting FASN in this context accordingly reduces entry into senescence. By contrast, our data suggest that, in lipogenesis-addicted cancer cells, endogenous FA synthesis is less wired as a generic energy-storage pathway and more wired as a constitutive requirement for maintaining the specialized membrane and organelle lipid environments that support the malignant phenotype. When ACC/FASN is blocked in cancer cells, they lose that lipogenic homeostasis and adapt by shifting into a glycerolipid-poor, acetyl-CoA/cholesterol-dependent senescent state. The ability of endogenous FASyn to perform diverse biological functions makes it mechanistically plausible that FASN activity can cause senescence in normal cells, yet its inhibition can trigger senescence in cancer cells. However, other studies in non-transformed human fibroblasts have shown that directly inhibiting ACC or FASN is itself sufficient to trigger a senescence-like program, including proliferation arrest, SA-β-gal positivity, DDR activation, ROS accumulation, and p38 MAPK signaling activation [116]. Thus, the relationship between lipogenesis and senescence is likely not binary but rather highly context- and stage-dependent. De novo lipogenesis may contribute to the development of some senescence programs; however, its sudden interruption can also act as a metabolic stressor that precipitates senescence. In this regard, FASTIS in cancer cells fits within a broader principle whereby lipogenic stress can be senogenic while contributing to the cancer-specific feature of an adaptive, cholesterol-rich state. Importantly, this convergence raises a translational caveat. If pharmacological ACC/FASN blockade provokes senescence-like states in normal cells, then FASTIS responses in non-malignant tissues could contribute to iatrogenic toxicity, as demonstrated for other forms of TIS [10–13]. Therefore, a key unresolved question is whether the low-inflammatory but persistent metabolic rewiring that characterizes FASTIS in cancer cells has counterparts in normal tissues systemically exposed to lipogenesis inhibitors.

The FASTIS-associated SASP secretome, which we termed FASASP, displayed much lower levels of pro-tumorigenic inflammatory components than the highly inflammatory secretomes induced by oxidative and DNA-damaging senogenic agents, such as bleomycin. Consequently, FASTIS cells secrete significantly lower amounts of pleiotropic pro-inflammatory SASP factors, including IL-6, CXCL8 (IL-8), MCP-1/CCR2, TNFα, and or CXCL1 (GROα). This low-inflammatory profile was mechanistically associated with limited activation of miR146a, a feedback regulator that is normally induced to prevent excessive inflammatory signaling [25, 94, 95]. Since NF-κB directly activates miR146a in response to senescence-related stimuli, such as LPS, TNFα, and IL-1β, and miR-146 dampens NF-κB activity and pro-inflammatory cytokine production [117, 118], these findings suggest that FASASP largely lacks an NF-kB-associated secretory phenotype (NASP). FASASP resembles the low-inflammatory SASPs induced by physiological hypoxia or CDK4/6is, such as palbociclib [17, 53, 92]. Importantly, except for IL-6, most factors significantly enriched in FASASP (86%) are linked to immune modulation. Thus, FASASP preserves the expression of cytokines that can enable anti-tumor immune responses. These include IL-18, which promotes T-cell expansion and differentiation of effector cells, and can enhance the tumoricidal activity of CAR-T cells [119]; MIP-3α/CCL20, which strongly attracts lymphocytes and dendritic cells [120]; IP-10, which is anti-angiogenic and immunomodulatory, and can enhance the antitumor effects of CAR-T cells [121]; and CX3CL1/fractalkine, which is a “*find me*” signal that recruits immune cells toward dying cells, and may serve as a biomarker of immunotherapy response [122]. Because the composition of the SASP critically determines whether TIS impairs or promotes immune clearance, further research is needed in additional ACC/FASN-overexpressing cancer models to define the specificity and therapeutic relevance of these immunomodulatory FASASP components. Overall, FASynis may represent a new class of senogenic drugs with immunotherapeutic value. By avoiding NF-kB-driven pro-tumorigenic inflammation while preserving immunomodulatory signals, FASynis may subject FASTIS cells to effective immunosurveillance, favoring their elimination by innate and adaptive immunity. At the same time, some FASASP factors could serve as circulating biomarkers of beneficial FASTIS activity in vivo and as useful readouts for novel lipogenesis-based immunosenotherapeutic strategies.

In line with recent studies indicating that senescent cells within residual disease can survive therapy by activating targetable immunomodulatory programs [65, 123, 124], we discovered that the FASTIS state renders breast cancer cells susceptible to inducible PD-L1 expression. Specifically, PD-L1 emerged as an IFNγ-induced and FASTIS-enhanced immunomodulatory response. In this regard, FASTIS resembles other mechanistically distinct TIS models, such as doxorubicin-induced senescence. These TIS states promote chromatin reorganization and increased accessibility, enabling expression of multiple immune checkpoints, including PD-L1, in residual senescent cancer cells after treatment [65]. Since sequential treatment with TIS inducers and PD-1/PD-L1 blockade increases T-cell accumulation and improves immune clearance [65, 68, 125], our findings support combining FASynis with anti-PD-L1 therapies in breast cancer treatment. Recent evidence also indicates that TIS cancer cells upregulate MHC-I antigen-presenting machinery and display an altered, highly immunogenic immunopeptidome [2, 3]. Given the growing importance of immune-mediated clearance as a partner to senescence-inducing therapies [126, 127], it is important to determine whether the immunopeptidome and surfaceome of FASTIS cells reveal extracellular vulnerabilities that could be exploited using neutralizing antibodies, antibody-drug conjugates, or CAR-T/CAR-NK cells as targeted FASTIS immunosenolytics [128, 129]. In parallel, inducible CRISPR/Cas9 systems that are activated only after FASTIS induction could help to uncover acquired vulnerabilities for developing FASTIS-targeted senolytics [130]. Finally, identifying FASTIS-specific biomarkers would be valuable for monitoring the burden of these cells in tumors, stratifying patients for senotherapies, and assessing treatment success by tracking their reduction over time.

A key requirement for using TIS in “one-two punch” strategies is that the collateral vulnerabilities acquired during the metastable senescent state induced by the first punch—whether chemotherapy, radiotherapy, targeted therapies, or now FASynis—must persist in a substantial fraction of cancer cells after the senescence-inducing treatment stops [13, 18, 21]. Under this framework, a second punch based on senotherapies, such as small molecule senolytics, PD-L1-targeting immunotherapies, or synthetic immune-cell approaches (e.g., cell-based immunotherapy, surfaceome-targeted antibodies), could selectively eliminate (FAS)TIS cells in the tumor. This would limit resistance, recurrence, and metastasis while clearing senescent cells from normal tissues and reducing treatment-related toxicity. Since senescence develops over time, TIS inducers, such as FASynis, and senotherapeutic agents can be administered sequentially. This approach has the potential to minimize overlapping toxicities, a phenomenon known as “synthetic lethality 2.0.” Even when TIS is transient, as with olaparib, the acquired liabilities of this temporary state can be exploited therapeutically through “one-two punch” approaches [70]. Early clinical and preclinical studies with FASyn-pathway inhibitors, including ACCi ND-646/ND-630 (firsocostat) and FASNi TVB-2640/TVB-3166, used continuous once-daily dosing schedules [39, 44, 131, 132]. These regimens can produce an immediate antitumor effect through senescence-associated growth arrest and promote the spread of the senescent phenotype to neighboring cells via the FASASP, thereby creating bystander sensitization to FASTIS-targeted senolytics or immunosenolytics. Although deep FASTIS appears to require sustained exposure to FASynis, senescent-cell accumulation takes at least 7 days in vitro. This supports intermittent “hit-and-run” schedules rather than necessarily daily or weekly dosing. Combining FASynis with other TIS inducers that reinforce cell-cycle arrest, such as palbociclib, might further reduce escape from senescence. However, because FASTIS may also favor resistance and relapse after drug withdrawal, the pharmacological elimination of FASTIS cells is mandatory to complement the therapeutic efficacy of FASyn-targeted cancer therapy and reduce the risk of cancer recurrence. Thus, FASTIS can be considered a targetable adaptive mechanism of breast cancer cells’ response to ACC/FASN inhibitors that can be therapeutically exploited as a collateral sensitivity using sequential combinations of pharmacological and immunological approaches.

We provide evidence that exploiting the acquired vulnerabilities of the FASTIS phenotype with a “one-two punch” senogenic-(immuno)senolytic strategy could offer a rational way to enhance the clinical value of FASynis (Fig. 11). Two independent studies of breast cancer patients treated with neoadjuvant chemotherapy regimens have identified TIS-associated biomarkers in 40% of post-treatment surgical specimens [133, 134], supporting the idea that FASynis could be tested as pro-senescence agents together with personalized (immuno)senolytic approaches in the neoadjuvant setting. Using BH3 profiling to assess proximity to the apoptotic threshold, we found that FASTIS cells appeared to display increased overall mitochondrial priming, though there was no clear BH3-profiled pattern of pro-survival adaptation. Nevertheless, FASTIS cells exhibited the previously observed increased dependence of TIS cancer cells on BCL-xL for survival [22–26, 96], as demonstrated by their greater sensitivity to the BCL-2/BCL-xL senolytic ABT-263/navitoclax and, particularly, to the BCL-xL-selective BH3 mimetics A-1331852. Notably, cancer cells that are already highly primed prior to senescence induction, such as FASN-overexpressing SKBR3 cells that exhibit robust baseline responses to BIM and PUMA, appear predisposed to becoming sensitive to BCL-xL-targeting BH3 mimetics post-senescence, irrespective of the senescence trigger [22]. Therefore, FASTIS cells may inherit and preserve pre-existing anti-apoptotic mitochondrial dependencies from their pre-senescent state [26]. Consistent with this, we previously reported that FASynis strongly induce pro-apoptotic BH3 proteins [45, 49], recapitulating the ability of BH3 mimetics to enhance mitochondrial cytochrome c release in ABT-263/A-1331852-sensitive TIS cells [22–26, 96]. Notably, the enhanced cytolytic response and accelerated extinction kinetics of FASTIS cells when interacting with cytokine-activated T cells suggest that FASTIS cells are more susceptible to either weak or infrequent immune-cell hits and/or more potently recruit immune cells. This supports our recently proposed role of lipogenic enzymes as novel tumor cell-intrinsic metabolic checkpoints that restrict T cell immunity [84]. Taken together, these findings suggest that FASTIS increases the susceptibility of breast cancer cells to BCL-2/BCL-xL-targeting BH3 mimetics and non-MHC-restricted cytotoxic lymphocytes. Finally, since ACC/FASN-driven lipogenesis is a pre-adaptive feature that helps breast cancer cells survive in the lipid-poor brain microenvironment and is already present in primary tumors [135–137], inducing FASTIS may be evaluated as a strategy to prevent brain metastasis in high-risk patients, especially those with triple-negative or HER2-positive disease.

## Conclusions

We identified FASTIS as a novel class of TIS triggered by the pharmacological blockade of de novo lipogenesis. Rather than merely killing lipogenesis-addicted breast cancer cells, clinically relevant ACC and FASN inhibitors can induce a metastable senescent state instead. Thus, targeted FASyn blockade redirects the utilization of acetyl-CoA and intracellular cholesterol handling into an adaptive senescent state, rather than simply causing a passive lipogenic arrest. This enables cancer cell survival and constitutes a plausible mechanism of adaptive escape. Notably, this cellular fate is not therapeutically inert as FASTIS cells acquire actionable vulnerabilities. Our findings expand the poorly understood biology of metabolic TIS and suggest that the clinical value of FASyn inhibitors can be maximized by actively targeting the liabilities created by the FASTIS intermediate cell fate. If clinically validated, this metabolic adaptation to targeted lipogenesis inhibition could be exploited therapeutically through rational “one-two punch” senogenic-(immuno)senolytic strategies.

## Materials and Methods

### Cell lines and culture conditions

The breast cancer cell lines SKBR3, MCF-7, MDA-MB-231, and BT-474 were obtained from the ATCC (Manassas, VA, USA). Cells were routinely expanded in Dulbecco’s modified Eagle’s medium (DMEM, Gibco) supplemented with 10% heat-inactivated fetal bovine serum (FBS; Linus), 1% L-glutamine, 1% sodium pyruvate, 50 IU/mL penicillin, and 50 μg/mL streptomycin. HAP1 and HAP1 *FASN-KO* cells (#HZGHC003700c006) were purchased from Horizon Discovery Ltd. (Cambridge, UK) and routinely grown in IMDM medium (Gibco) supplemented with 10% FBS, 2 mmol/L L-glutamine, and 100 IU/mL penicillin/streptomycin. JIMT-1 cells were obtained from the German Collection of Microorganisms and Cell Culture (Braunschweig, Germany) and grown in Dulbecco’s modified Eagle’s medium (DMEM) supplemented with 10% heat-inactivated FBS, 1% L-glutamine, 1% sodium pyruvate, 50 IU/mL penicillin, and 50 μg/mL streptomycin. CRISPR-based JIMT-1/*FASN* KO cells were generated using an Edit-R pre-designed all-in-one lentiviral sgRNA as described elsewhere [84].

Cells were grown at 37 °C in a humidified atmosphere containing 5% CO_2_ and were in the logarithmic growth phase at the start of the experiments. Cell lines were authenticated by STR profiling, performed by the manufacturer and confirmed in-house at the time of purchase, in accordance with ATCC guidelines. Cells were passaged by starting a low-passage cell stock every month for up to 2–3 months after resuscitation. Cell lines were screened for mycoplasma contamination using a PCR-based method prior to experimentation and were intermittently tested thereafter.

### Drugs and reagents

TVB-3166 (#S3576), ND-646 (#S8377), Doxorubicin (#S1208), PD-0332991/palbociclib (#S1116), Ac-CoA Synthase Inhibitor 1 (S8588), rosuvastatin (#S2169), ABT-263/navitoclax (#S1001), and A1331852 (#S7801) were purchased from Selleck Chemicals LLC (Houston, TX, USA). MLN8237/alisertib (#331-10890-1) and bleomycin sulfate (#331-11727-3) were purchased from RayBiotech Inc. (Norcross, GA, USA). C75 (#270-286-M005) was purchased from Alexis Biochemicals (San Diego, CA, USA). WX-8 (#SML3288) was purchased from Merck KGaA (Darmstadt, Germany). Soraphen A purified from the myxobacterium *Sorangium cellulosum* was kindly provided by Drs. Klaus Gerth and Rolf Jansen (Hemholtz Zentrum für Infektionsforschung GmbH, Braunschweig, Germany). Compounds were dissolved in DMSO, and stored as stock solutions at −20 °C in the dark until utilization.

AlamarBlue™ Cell Viability Reagent (#DAL1100), Lysotracker^®^ Red DND-99 (Cat. #L7528), JC-1 dye (mitochondrial membrane potential probe; #T3168), and PageRuler™ Plus Prestained Protein Ladder (#26619) were purchased from Thermo Fisher Scientific Inc. (Waltham, MA, USA). Antibodies to p21^WAF1/CIP1^ (12D1; #2947), PD-L1 (E1L3N^®^ XP^®^ Rabbit mAb #13684), p70 S6 Kinase (#9202), and phospho-p70 S6 Kinase^Thr421/Ser424^ (#9204), and senescence β-galactosidase staining kit (#9860) were purchased from Cell Signaling Technology, Inc. (Danvers, MA, USA). Antibodies to phospho-RB1 (Ser807/811; #30376-1-AP) and β-actin (#66009-1-Ig) were purchased from Proteintech Group, Inc. (Rosemont, IL, USA). Filipin complex ready-made solution (#SAE0088) and the Cholesterol Quantification Assay kit (#CS0005) were purchased from Sigma-Aldrich (St. Louis, MO, USA).

### Senescence-associated β-galactosidase activity (SA-β-Gal) staining

Cellular β-galactosidase activity was detected using the Senescence β-Galactosidase Staining Kit (Cell Signaling Technology, Danvers, MA, USA) according to the manufacturer’s instructions. To quantitatively assess senescence induction, cells were seeded into 6-well plates and treated with senescence inducers for 7 days (without drug refreshment or with FASynis refreshment on day 3, as specified) before SA-β-gal staining. The total number of cells was counted from five images/well from the same experiment, and at least 1000 cells were counted per condition.

### IncuCyte® assays

Cells (2000/well) were seeded into 96-well plates and cultured as indicated. Images were auto-collected using the IncuCyte® ZOOM incubator (IncuCyte® S3 Live-Cell Analysis Platform; Essen BioScience, Ann Arbor, MI, USA). Data analysis was made using the Incucyte software package (Essen BioScience, Ann Arbor, MI, USA).

### Therapy-induced senescence

Cells were treated with the following concentrations of palbociclib (Palbo), bleomycin (Bleo), doxorubicin (Doxo), alisertib (Ali), TVB-3166 (TVB), and ND-646 (ND) concentrations: MCF-7 and MDA-MB-231, 0.5 μmol/L TVB, 0.5 μmol/L ND, 1 μmol/L Palbo, 20 μmol/L Bleo, 50 nmol/L Doxo, 0.5 μmol/L Ali; BT-474, 0.25 μmol/L TVB, 0.25 μmol/L ND, 1 μmol/L Palbo, 10 μmol/L Bleo, 20 nmol/L Doxo, 0.5 μmol/L Ali; SKBR3, 0.25 μmol/L TVB, 0.25 μmol/L ND, 1 μmol/L Palbo, 10 μmol/L Bleo, 10 nmol/L Doxo, 0.5 μmol/L Ali. Cells were treated 24 h after seeding and drug was refreshed every 3 days for up to 7 days.

### Quantitative real-time polymerase chain reaction (qRT-PCR)

Total RNA was extracted from cells using RNA Plus Kit (Macherey-Nagel, Germany) following the manufacturer’s instructions. Two micrograms of total RNA were reverse-transcribed into cDNA using High-Capacity cDNA Reverse Transcription Kit (Life Technologies, Carlsbad, CA, USA). RNA concentration and quality were determined using a NanoDrop™ ND-1000 spectrophotometer (NanoDrop Technologies, USA).

To evaluate the senescence-associated upregulation of CDK inhibitors and immune checkpoints, the abundance of CDKN1A/p21 (Hs00355782_m1), CDKN1B/p27 (Hs00153277_m1), CDK1NC/p57 (Hs00175938_m1), CDKN2A/p16 (Hs00923894_m1), and CD274/PD-L1 (Hs01125301_m1) relative to the housekeeping genes 18S (Hs99999901_s1) and PPIA (Hs99999904_m1) was evaluated using an Applied Biosystems QuantStudio^TM^ Flex PCR System with an automated baseline and threshold cycle detection. The transcript abundance was calculated using the ΔΔCt method and presented as relative quantification (RQ).

For validation of RNA-seq results, gene expression was assessed using TaqMan Custom Array Plates (Thermo Fisher Scientific) containing predesigned assays for selected genes, namely: *ABCG1* (Hs00245154_m1), *ACSS2* (Hs01122829_m1), *ANGPTL4* (Hs01101123_g1), *CCL22* (Hs01574247_m1), *CD63* (Hs01041238_g1), *CILP2* (Hs00541922_m1), *CX3CL1* (Hs00171086_m1), *FDPS* (Hs01578769_g1), *FSD1* (Hs00976143_m1), *GDF15* (Hs00171132_m1), *GJA5* (Hs00270952_s1), *HMGCS2* (Hs00985427_m1), *IRF9* (Hs00196051_m1), *LCN2* (Hs01008571_m1), *MED12L* (Hs00326378_m1), *MSMO1* (Hs00932159_m1), *MVD* (Hs00964565_m1), *OAS1* (Hs00973635_m1), *OAS2* (Hs00942643_m1), *OLAH* (Hs00217864_m1), *PLSCR1* (Hs01062171_m1), *S100P* (Hs00195584_m1), *SCD* (Hs01682761_m1), *SDR16C5* (Hs00977334_m1), *ST6GALNAC2* (Hs01032565_m1). *PPIA* (Hs04194521_s1), *GAPDH* (Hs99999905_m1), and *GUSB* (Hs99999908_m1) were used as reference genes. Data were analyzed using Thermo Fisher Cloud software, and relative expression was calculated using the ΔΔCt method and expressed as log2 fold-change.

### Immunoblotting

Immunoblotting, including cell lysis, sodium dodecyl sulfate polyacrylamide gel electrophoresis (SDS-PAGE), transfer, and incubation with primary and secondary antibodies, was performed as previously described [24, 25]. Chemiluminescence detection images were acquired using a ChemiDocMP imaging system (Bio-Rad). Uncropped, full-length western blots were merged with the corresponding protein size ladder. They are available in the Supplementary Material.

### RNA sequencing (RNA-seq) analysis

Total RNA was extracted from cells using the RNA Plus Kit (Macherey-Nagel, Germany) according to the manufacturer’s instructions. RNAseq and bioinformatics analysis were outsourced to Macrogen Inc (Republic of Korea). Briefly, RNA-seq libraries were prepared using the Illumina^®^ TruSeq^®^ Stranded Total RNA Sample Preparation Kit (Illumina, Inc., San Diego, USA) according to the manufacturer’s recommendations. Samples were sequenced on Illumina’s NovaSeq 6000 system using 2 × 150 bp paired-end reads. Reads were aligned to the human reference genome GRCh37 using HISAT2 (v2.1.0) [138], and transcript assembly was performed using StringTie (v2.1.3b) [139]. Differentially expressed gene (DEG) analysis was performed on comparative pairs using DESeq2 [140].

### Enrichment analysis

Gene set enrichment analysis based on the Gene Ontology (GO) database was performed with the significant DEG list using the g: Profiler tool (https://biit.cs.ut.ee/grolifer/) [141]. The g: Profiler tool performs statistical enrichment analysis to find overrepresentation of information from GO terms, biological pathways, regulatory DNA elements, human disease gene annotations, and protein-protein interaction networks.

The web-based gene set enrichment analysis platform Enrichr [142] was used to perform enrichment analysis of either common genes up- or downregulated after TVB-3166 and ND-646 treatment or genes that were exclusively up- and downregulated after TVB-3166 and ND-646 treatment compared to all other TIS inducer treatments.

Gene set enrichment analysis (GSEA) was performed using the web-based GenePattern platform [143]. The human hallmark gene collection was downloaded from the Human Molecular Signatures Database (MSigDB) [144]. Gene sets with FDR *q* < 0.1 were considered to be significant.

### Metabolomics and lipidomics

Untargeted metabolomic and lipidomic analyses of SKBR3 FASTIS cells and their matched proliferative controls (*n* = 3 biological replicates/each) were outsourced to oloBion (Barcelona, Spain). OloBion is an omics bioscience laboratory equipped with mass spectrometry platforms for profiling small-molecules and lipids (https://www.olobion.ai). To prepare the samples, the cellular material was homogenized in cold methanol. Then, methyl tert-butyl ether (MTBE) containing an internal standard was added. After shaking, 10% methanol was added. The samples were shaken again, then centrifuged to achieve phase separation.

For metabolomics, two aliquots of the lower aqueous phase were processed using two different chromatographic methods. For hydrophilic interaction liquid chromatography (HILIC), the samples were evaporated to dryness, reconstituted in acetonitrile/water containing internal standards, shaken, and centrifuged. For reversed-phase liquid chromatography (RPLC), the samples were mixed with isopropanol/acetonitrile (1:1), shaken, and centrifuged. Then, the samples were evaporated and resuspended in 5% methanol containing internal standards, followed by final centrifugation. Separation of polar metabolites was performed using Waters ACQUITY UPLC BEH Amide and ACQUITY UPLC HSS T3 50 mm columns, both of which were maintained at 45 °C, on an Agilent 1290 Infinity UHPLC system coupled to a ZenoTOF 7600 mass spectrometer (Sciex). Data were acquired in both positive and negative electrospray ionization (ESI) modes to maximize metabolite coverage.

For lipidomics, the upper organic phase was collected and evaporated. Then, it was resuspended in methanol containing an internal standard and shaken and centrifuged before LC-MS analysis. Lipid separation was performed on a Waters ACQUITY UPLC BEH C18 50 mm column, which was maintained at 65 °C. The analysis was carried out using an Agilent 1290 Infinity UHPLC coupled to a ZenoTOF 7600 mass spectrometer (Sciex), with acquisition in both positive and negative ESI modes.

Raw mass spectrometry data were processed using oloMAP, a proprietary, AI-assisted platform designed for high-throughput data processing and compound annotation. The workflow incorporated an in-house adaptation of MS-DIAL 4.9 to automatically detect and align features, followed by a multistage annotation pipeline based on accurate mass, retention time and MS/MS spectral matching against validated reference libraries. For metabolomics, annotation was supported by a custom spectral database comprising approximately 1.3 million spectra corresponding to over 60,000 unique chemical entities [145]. For lipidomics, lipid species were identified using MS-DIAL 5.5. LipidDB and in-house libraries containing 5.2 million spectra mapping to 2.5 million unique lipid structures [146]. In both workflows, machine learning-based retention-time prediction was used to filter and rank candidate annotations. For lipidomics, species-level calls underwent an additional validation and relative quantification step using authentic standards spanning 14 lipid classes. Lipid classes not represented by these standards were normalized to a C16 ceramide standard, which enabled consistent comparison across samples. Statistical analyses and data visualization were performed using the oloMAP Portal platform.

### Filipin staining

A ready-made Filipin complex solution (5 mg/mL in DMSO) was diluted to a working concentration of 0.05 mg/mL in phosphate-buffered saline (PBS) supplemented with 10% FBS. Cells were washed three times with phosphate-buffered saline (PBS) and fixed with freshly prepared 3% paraformaldehyde (PFA) for 1 hour at room temperature. After fixation, cells were washed three times with PBS and incubated with 1.5 mg/mL glycine in PBS for 10 min at room temperature to quench residual PFA. Cells were then incubated with the filipin working solution for 2 h at room temperature in the dark. After staining, cells were washed three times with PBS and maintained in PBS for imaging. Fluorescence imaging was performed using a fluorescence microscope equipped with a UV filter set. All filipin-containing solutions were protected from light throughout the procedure, and filipin fluorescence was imaged promptly due to its rapid photobleaching properties.

### Cholesterol quantification assay

Total cholesterol levels were quantified using the Cholesterol Quantification Assay Kit (Sigma-Aldrich) according to the manufacturer’s instructions. Briefly, cells were harvested after the indicated treatments, counted, and pelleted. Lipids were extracted by adding 200 µL of chloroform:isopropanol:IGEPAL^®^ CA-630 (7:11:0.1, v/v/v) to each pellet. Samples were then centrifuged at 13,000 × g for 10 min to remove insoluble material. The organic phase was transferred to a new tube and incubated at 50 °C for approximately 30 min to allow chloroform to evaporate. Residual solvent was then removed using a vacuum concentrator until the lipids were completely dry. Dried lipids were then resuspended in 200 µL of assay buffer and vortexed until a homogeneous solution formed. Cholesterol levels were then measured using the fluorometric assay format according to the manufacturer’s protocol. Samples and standards were incubated with the reaction mix in a black 96-well plate, protected from light. Fluorescence was measured using a Cytation 5 microplate reader (Ex/Em 535/587 nm). Cholesterol concentrations were determined from a standard curve and normalized to cell number.

### Secretome

The qualitative and quantitative composition of TIS-associated secretomes in terms of cytokines, chemokines, and growth factors was characterized using multiplex bead-based immunoassays Luminex^®^. Briefly, cell culture supernatants from non-senescent (proliferative controls) and cancer cells rendered senescent with mechanistically different TIS inducers as described above were collected in strict parallel and separated into two sets. One set was analyzed with an *in-house* Luminex^®^ platform (Luminex^®^ MAGPIX, XMAP technology) using the Human Cyto/Chemo/GF Panel A 38 Plex Kit (HCYTA-60K-PX38, Millipore) according to the manufacturer’s instructions. Another set was sent to a third-party commercial laboratory (Eve Technologies, Canada) to perform the Cytokine/Chemokine 96-Plex Discovery Assay^®^ Array (HD96).

### Generation of miR146a-eGFP reporter-expressing breast cancer cells

Viral particles were generated in 293 T cells by co-transfecting the PHAGE-PmiR-146a-GFP-PGK-puro plasmid with the pCMV-VSV-G/ pCMV-dR8.2 dvpr 3rd generation lentivirus packaging system. 293 T cells were transiently transfected to generate lentiviral supernatants, and SKBR3 cells were infected with lentiviral supernatants at 8 μg/mL Polybrene. After 48 h, medium containing 10 μg/mL puromycin was added for another 48 h to generate miR146a-EGFP reporter-containing SKBR3 cells.

### miR146a-EGFP flow cytometry

To measure miR146a induction as a surrogate marker of response to NF-κB-related SASP, miR146A-eGFP reporter-containing SKBR3 cells were rendered senescent as described above, harvested, and resuspended in 300 μL of medium. eGFP positivity was determined using a laser excitation wavelength of 488 nm (FITC channel) on a BD Accuri C6 flow cytometer (BD Biosciences, San Jose, CA, USA). Data were analyzed using FCS Express 7 software (De Novo™ Software, Pasadena, CA, USA).

### BH3 profiling

The JC-1 plate-based BH3 profiling was performed according to the original procedure provided by the Letai lab at the Dane-Farber Cancer Institute (https://letailab.dana-farber.org/bh3-profiling.html). Briefly, BIM, BID, BMF, PUMA, BAD, HRK, NOXA, and PUMA peptides (0.01/0.1–100 μmol/L), alamethicin (25 μmol/L), and CCCP (10 μmol/L) were added to JC-1 MEB staining solution (150 mmol/L mannitol, 10 mmol/L HEPES-KOH, 50 mmol/L KCl, 0.02 mmol/L EGTA, 0.02 mmol/L EDTA, 0.1% BSA, 5 mmol/L succinate, pH 7.5) in a black 384-well plate. Single cell suspensions from proliferative and 7-day TIS cell cultures were prepared in JC-1-MEB buffer, including 0.0025% digitonin, and kept at RT for 10 min to allow for cell permeabilization and dye equilibration. After adding 2–3 ×10^4^ cells/well to the 384-well plate, fluorescence will be measured at 590 nm emission/545 nm excitation using a microplate reader at 30 °C every 15 min for a total of 3 h to obtain kinetic traces for each peptide. Percent depolarization was calculated by normalization to the AUC of the solvent-only control (DMSO, % depolarization) to generate the final profiles.

### Cell viability and senolytic index

Cell viability was measured using alamarBlue™ assays. The non-toxic, cell-permeable alamarBlue™ (resazurin) reagent is an oxidized form of a redox indicator that is blue in color and non-fluorescent. Upon entering living cells, the reagent is reduced to resorufin, which changes color from blue to red and becomes fluorescent. The oxidized environment upon loss of cell viability maintains the non-fluorescent, blue color of resazurin.

To calculate senolytic indexes, untreated (proliferative) and FASTIS SKBR3 counterparts were harvested, reseeded in 96-well plates (8000 cells/100 μL/well) and exposed to graded concentrations of the statin rosuvastatin or BH3 senolytics for an additional 3 to 5 days, as specified. Cells were incubated with the alamarBlue solution (10 μL/well) at 37 °C for 4 h and the increase in fluorescence signal was measured using a fluorescence detector. The senolytic index was defined as the ratio between the inhibitory concentration 50 (IC_50_) values of proliferative cells and the IC_50_ values of ND-646- and TVB-3166-induced TIS cancer cells. The IC_50_ values were defined as the concentration of drug that produced a 50% reduction in control fluorescence (by interpolation).

### Cytokine-activated T cells

To obtain cytokine-activated T cells (CATs), human peripheral blood mononuclear cells (#70025) were cultured in ImmunoCult-XF™ T cell expansion medium (#10981) containing ImmunoCult-XF™ Human CD3/CD28/CD2 T cell activator (#10971) (all from StemCell Technologies, Vancouver, BC, Canada), and 10 ng/mL IL-2 (#200-02, PeproTech, Rocky Hill, NJ) for 1 week according to the manufacturer’s instructions. Cell-based immunotherapy assays with CATs were performed in the presence of anti-CD3 antibody (100 ng/mL; #16-0037; eBioscience, Thermo Scientific) and IL-2 (10 ng/mL).

### Crystal violet cell killing assay

Cells were seeded in 12-well plates at different densities per well. After an overnight attachment period, cells were exposed to different ratios of CATs for 48 h. Untreated and CAT-treated cells were washed twice with cold PBS and incubated with 0.5% (*w/v*) crystal violet solution in 25% methanol for 20 min. The methanol was aspirated, and the cells were incubated with 0.5% crystal violet solution in 25% methanol for 20 min. The cells were washed several times and dried overnight.

### Generation of nuclight-labeled breast cancer cells

SKBR3 breast cancer cells stably expressing green or red nuclear labeling were obtained after lentiviral transduction with the Incucyte^®^ Nuclight lentivirus (#4475 and #4476, respectively, Sartorius) followed by antibiotic selection. Briefly, cells at 40% confluence were transduced at a multiplicity of infection (MOI) of 1 in diluted media containing 8 μg/mL polybrene (MerckMillipore). The next day, the media were removed and replaced with fresh media containing 2 μg/mL of puromycin and cultured for 7 days for selection.

Mixed cultures of green (proliferative) and red nuclei (senescent) cancer cells in 96-well plates and 24 h later were treated with either BH3 mimetics or anti-CD3/CD28-activated PBMCs. The assay plates were placed into the IncuCyte^®^ Live-Cell Analysis System and scheduled for 4-hour repeat scanning using the Cell-by-Cell Analysis Software to detect the death of target cells (red cells for senescence-targeted senolysis).

### Statistical analysis

All cell-based observations were confirmed by at least three independent experiments, each performed in triplicate for every cell line and condition. Data are expressed as mean ± SD. Bar graphs, curves, and statistical analyses were generated using GraphPad Prism 10 (GraphPad Software, San Diego, CA). Ridge-density plots, box plots, and volcano plos were used to describe metabolomic and lipidomic data. ANOVA was used to compare means of ≥3 groups, and Dunnett’s multiple contrasts were used to test for individual differences in case of significant F values. *P* values < 0.05 were considered to be statistically significant. All statistical tests were two-tailed.

## Supplementary information

## Data Availability

All of the data sets used in the present study are available from the corresponding authors upon reasonable request. Accession number for the RNA-seq data reported in this paper is GEO: GSE292003 (https://www.ncbi.nlm.nih.gov/geo/query/acc.cgi?acc=GSE292003).
