# Supplementary material for: Fatty acid synthesis therapy-induced senescence (FASTIS) in cancer cells
Source: Cell Death Dis. 2026 Jun 17;17(1):770. doi: 10.1038/s41419-026-08992-8 (PMC13538499; doi:10.1038/s41419-026-08992-8)
Supplement: Supplementary file 1 [file 41419_2026_8992_MOESM1_ESM.docx]

**Fatty acid synthesis therapy-induced senescence (FASTIS) in cancer cells**

Supplementary Information

**Figure S1. Fatty acid synthesis and therapy-induced senescence (TIS). A.** *Top.* Activation of *de novo* lipogenesis is an established metabolic hallmark of breast cancer. FASyn acts as a source of structural components and signaling molecules that are essential for the proliferation and survival of cancer cells [1–3]. FASyn serves also as a multifaceted signaling hub that integrates internal metabolic states and external (environmental/therapeutic) signals to drive breast cancer initiation, therapeutic resistance, and metastatic progression [4,5]. FASyn is beginning to be understood as a sophisticated and targetable signalome that regulates multiple aspects of breast cancer pathophysiology, including phenotypic plasticity, evasion of cell death, immunoresistance, and site-specific metastatic tropism. Aberrant upregulation of FASyn enzymes has been associated with poor prognosis in several tumors including breast cancer. In breast cancer, FASyn is required for the formation of breast cancer metastases in the lipid-poor environment of the brain. *Bottom.* Pharmacological inhibition of ACC/FASN enzymes limits the committed catalytic steps of *de novo* FASyn, regardless of the tumor cells’ nutritional/metabolic sources. Despite challenges related to efficacy, selectivity and safety, several new classes of synthetic ACC and FASN inhibitors have entered clinical development and may form the basis of a new class of metabolic therapeutics for cancer treatment. ND-646 interacts with the biotin carboxylase domain of ACC to prevent its activating dimerization. TVB-2640/denifanstat is a reversible FASNi that blocks NADPH consumption in the FASN β-ketoreductase domain. **B.** We evaluated the ability of the ACCi ND-646 and the FASNi TVB-3166 (an *in vitro* analog of the clinical-grade TVB-2640) to activate a senescence-like phenotype to test the hypothesis that therapy-induced senescence (TIS), a well-known phenomenon driven by oxidative (bleomycin), genotoxic (doxorubicin), replicative (palbociclib), and mitotic (alisertib) stress, can also be induced by targeted inhibition of *de novo* lipogenesis. We comprehensively characterized this fatty acid synthesis therapy-induced senescence (FASTIS) state by assessing the following: senescence-associated (SA) cytomorphological remodeling, SA beta-galatosidase (SA-β-gal) activity, expression of cell cycle arrest and immune escape biomarkers (p21, p57, p27, phospho-Rb, PD-L1), mRNA-based senescence transcriptional signatures, SA metabolomic and lipidomic remodeling, SA secretory phenotype (SASP), mitochondrial apoptotic priming, and sensitivity to (immuno)senolytics.

**Figure S2. Antiproliferative and clonogenic effects of FASN and ACC inhibition across breast cancer cell lines**. The figure illustrates plaque formation (**A**) and clonogenic assays (**B**), which demonstrate the impact of varying concentrations (0.05–0.5 μmol/L) of the FASN inhibitor TVB-3166 (TVB) and the ACC inhibitor ND-646 (ND) on MDA-MB-231, MCF7, and SKBR3 breast cancer cells. For the plaque formation assays, MDA-MB-231 and MCF7 cells were seeded at 20,000 cells/well and SKBR3 cells at 40,000 cells/well. Treatment was initiated 24 h after seeding, refreshed on day 3, and the plates were fixed and stained with crystal violet on day 5. For the clonogenic assays, MDA-MB-231 and MCF7 cells were seeded at 250 cells/well and SKBR3 cells at 500 cells/well. Treatment was initiated 24 h after seeding, refreshed on day 3. Colonies were stained with crystal violet on day 10. The inhibitors were more effective against the lipogenic SKBr3 and MCF7 models than against MDA-MB-231 cells. Images are representative of three independent experiments.

**Figure S3.** **SA-β-gal staining in breast cancer cell models treated with first-generation FASyn inhibitors.** Representative images (n=3) of SA-β-gal staining in SKBR3 and BT-474 breast cancer cells treated with the ACC inhibitor soraphen A (100 nmol/L) and the FASN inhibitor C75 (5 μg/mL). Scale bars, 200 μm.

**Figure S4.** **Principal component analysis (PCA) plot of the similarity between TIS transcriptomes using log transformed values and two principal components.** This approach maximizes the preservation of the data variability and allows the identification of any outlier samples or similar expression patterns between groups of samples.

**Figure S5. Functional annotation analysis of the FASTIS-associated transcriptome (I).** Dot plots show the top 20 most significant GO terms with term size greater than 10 and less than 500 in each category (biological process, cellular component, and molecular function) for ND-646 TIS (*left panels*) and TVB-3166 TIS (*right panels*). GO terms were selected if their adjusted *p*-value (adj. *p*-value) was less than 0.05 and the term size was between 10 and 500. GO terms were then sorted by adj. p-value and the top 20 most significant GO terms were selected. Finally, they were arranged in descending order by GeneRatio. X-axis and Y-axis are GeneRatio and GO terms, respectively. Dot size is the intersection size.

**Figure S6. Functional annotation analysis of the ND-646 TIS-associated transcriptome (II).** GO term histograms of the intersection genes in the ND-646 TIS-associated transcriptome. The top 10 GO terms with the smallest adjusted *p*-values in each category (biological process, cellular component, and molecular function) are shown.

**Figure S7. Functional annotation analysis of the TVB-3166 TIS-associated transcriptome.** GO term histograms of the intersection genes in the ND-646 TIS-associated transcriptome. The top 10 GO terms with the smallest adjusted *p*-values in each category (biological process, cellular component, and molecular function) are shown.

**Figure S8. Comparative functional annotation analysis of TIS-associated transcriptomes.** Dot plots show the top 20 most significant GO terms with term size greater than 10 and less than 500 in each category (biological process, cellular component, and molecular function) for all the TIS transcriptomes in one or more comparisons. GO terms were selected if their adjusted *p*-value (adj. *p*-value) was less than 0.05 and the term size was between 10 and 500. GO terms were then sorted by adj. p-value and the top 20 most significant GO terms were selected. Finally, they were arranged in descending order by GeneRatio. X-axis and Y-axis are TIS phenotypes and GO terms, respectively. Dot size is Gene Ratio.

**Figure S9. Representative SA-β-gal staining of HAP1 and HAP1 *FASN-KO* cells following TVB-3166 treatment.** Representative brightfield images of SA-β-gal staining in parental HAP1 cells and *FASN KO* isogenic derivatives under untreated conditions or after TVB-3166 treatment. Scale bars, 200 μm.

**Figure S10. Chemical superclass annotation of differential metabolites in ND-646- and TVB-3166-induced TIS cells. A.** Pie charts showing the distribution of annotated differential metabolites according to ClassyFire Superclass (*left*) and NPClassifier Superclass (*right*) [6,7]. **B.** Bubble plots summarizing superclass-level changes in ND-646-induced TIS (*left*) and TVB-3166-induced TIS (*right*). The upper row shows ClassyFire Superclass categories and the lower row shows NPClassifier Superclass categories. The x-axis indicates the mean –log_10_(*p*-value), the y-axis indicates mean log2(fold change), and bubble size represents the number of metabolites assigned to each superclass. Colors denote the corresponding superclass categories shown in the legends.

**Figure S11. Multivariate lipidomic analysis and lipid-category enrichment in ND-646- and TVB-3166-induced FASTIS cells.**
*Top left,* **principal component analysis (PCA)** score plot of lipidomic profiles from **proliferative**, **ND-646-induced TIS** and **TVB-3166-induced TIS** cells. *Top right,* **partial least squares discriminant analysis (PLS-DA)** score plot of the same sample groups. *Bottom left*, bubble plot showing **chemical enrichment** results for **ND-646-induced TIS** compared to proliferative cells. *Bottom right,* bubble plot showing **chemical enrichment** results for **TVB-3166-induced TIS** compared to proliferative cells. In the enrichment plots, the x axis indicates mean **−log10(*p*-value),** the y axis indicates mean **log2(fold change)**, bubble size represents the number of lipid species in each category, and colors denote the indicated **lipid categories**.

**Figure S12. Representative differentially abundant lipid species in ND-646- and TVB-3166-induced FASTIS cells.**
Box plots showing the **top decreased**/**increased** lipid species ranked by fold-change with ***p* < 0.0001** across **proliferative**, **ND-646-induced TIS,** and **TVB-3166-induced TIS** cells. Individual lipid species are shown with their corresponding annotations and normalized abundance values, which are expressed as **ng/10^6^ cells**. The *y*-axis indicates **lipid intensity**. Dots represent individual samples, and center lines indicate the median. Boxes indicate the interquartile range, and whiskers indicate the range (n=3 for proliferative and ND-646 FASTIS cells and n=2 for TVB-3166 FASTIS cells).

**Figure S13. Lysosomal signal quantification in FASTIS cells generated in the absence/presence of the PIKfyve inhibitor WX-8.** *Top,* representative **bright-field images** of **untreated**, **TVB-3166-induced TIS** and **ND-646-induced TIS** cells cultured in the absence (**0 μmol/L**) or presence (**1 μmol/L**) of **WX-8**. *Bottom left,* representative **phase-contrast/LysoTracker Red overlay images** of the same experimental groups. **Red fluorescence corresponds to LysoTracker staining of lysosomes.** *Bottom right*, bar plot of the **RCU/%** values (mean ± SD) for **untreated** and **TVB-3166-** or **ND-646-treated** cells at **0 μmol/L** or **1 μmol/L WX-8**. **RCU/%** denotes the **red mean intensity object average normalized to phase area confluence**. Scale bars, **200 μm** in the top panels and as indicated in the bottom panels.

**References (Supplementary Material)**

1. Menendez JA, Lupu R. [Fatty acid synthase and the lipogenic phenotype in cancer pathogenesis.](https://pubmed.ncbi.nlm.nih.gov/17882277/) Nat Rev Cancer. 2007;7:763–77
2. Koundouros N, Poulogiannis G. [Reprogramming of fatty acid metabolism in cancer.](https://pubmed.ncbi.nlm.nih.gov/31819192/) Br J Cancer. 2020;122:4–22.
3. Röhrig F, Schulze A. [The multifaceted roles of fatty acid synthesis in cancer.](https://pubmed.ncbi.nlm.nih.gov/27658529/) Nat Rev Cancer. 2016;16:732–49.
4. Vanauberg D, Schulz C, Lefebvre T. [Involvement of the pro-oncogenic enzyme fatty acid synthase in the hallmarks of cancer: a promising target in anti-cancer therapies.](https://pubmed.ncbi.nlm.nih.gov/36934087/) Oncogenesis. 2023;12:16.
5. [Menendez JA, Cuyàs E, Encinar JA, Vander Steen T, Verdura S, Llop-Hernández À, López J, Serrano-Hervás E, Osuna S, Martin-Castillo B, Lupu R Fatty acid synthase (FASN) signalome: A molecular guide for precision oncology.](https://pubmed.ncbi.nlm.nih.gov/38158755/) Mol Oncol. 2024;18:479–516.
6. Djoumbou Feunang Y, Eisner R, Knox C, Chepelev L, Hastings J, Owen G, Fahy E, Steinbeck C, Subramanian S, Bolton E, Greiner R, Wishart DS. ClassyFire: automated chemical classification with a comprehensive, computable taxonomy. J Cheminform. 2016;8:61.
7. Kim HW, Wang M, Leber CA, Nothias LF, Reher R, Kang KB, van der Hooft JJJ, Dorrestein PC, Gerwick WH, Cottrell GW. [NPClassifier: A Deep Neural Network-Based Structural Classification Tool for Natural Products.](https://pubmed.ncbi.nlm.nih.gov/34662515/) J Nat Prod. 2021;84:2795–807.
