# Supplementary material for: Fatty acid synthesis therapy-induced senescence (FASTIS) in cancer cells
Source: Cell Death Dis. 2026 Jun 17;17(1):770. doi: 10.1038/s41419-026-08992-8 (PMC13538499; doi:10.1038/s41419-026-08992-8)
Supplement: Supplementary file 2 [file 41419_2026_8992_MOESM2_ESM.docx]

**Fatty acid synthesis therapy-induced senescence (FASTIS) in cancer cells**

**Uncropped original WBs**

**Figure 1D**

**Figure 1D**

**Figure 1D**

**Figure 7B**

**55**

**35**

**70**

**100**

**55**

**35**

**70**

**100**

**55**

**35**

**70**

**100**

**55**

**35**

**70**

**100**

**55**

**35**

**70**

**100**

**55**

**35**

**70**

**100**

**mbr1**

**mbr2**

**p-p70 S6K**

**p70 S6K**

**β-actin**

**β-actin**

CT

TVB

ND

CT

TVB

ND

CT

TVB

ND

CT

TVB

ND

SKBr3

MCF7

SKBr3

MCF7

CT

TVB

ND

CT

TVB

ND

SKBr3

MCF7

CT

TVB

ND

CT

TVB

ND

SKBr3

MCF7
